# The Association Between Vitamin D Status and Depression Severity in Adults with Major Depressive Disorder: A Cross-Sectional Study

**DOI:** 10.3390/ijms27188192

**Published:** 2026-09-15

**Authors:** Andreea Roșian, Otilia Micle, Luciana Dobjanschi, Laura Grațiela Vicaș, Mariana Eugenia Mureșan, Camelia Maria Dindelegan, Rita Ioana Platona, Roxana Georgeta Pali, Darius Gabriel Florea, Alin Cristian Teușdea, Eleonora Marian

**Affiliations:** 1 Doctoral School of Biomedical Sciences, Faculty of Medicine and Pharmacy, University of Oradea, 410087 Oradea, Romania; rosian.andreea@student.uoradea.ro (A.R.); omicle@uoradea.ro (O.M.); dobjanschil@uoradea.ro (L.D.); mmuresan@uoradea.ro (M.E.M.); emarian@uoradea.ro (E.M.); 2 Department of Preclinical Disciplines, Faculty of Medicine and Pharmacy, University of Oradea, 410087 Oradea, Romania; 3 Department of Pharmacy, Faculty of Medicine and Pharmacy, University of Oradea, 410087 Oradea, Romania; 4 Department of Psychology, Faculty of Socio-Human Sciences, University of Oradea, 410087 Oradea, Romania; camelia.dindelegan@didatic.uoradea.ro; 5 Department of Neuroscience, Faculty of Medicine and Pharmacy, University of Oradea, 410087 Oradea, Romania; 6 Psychiatry Department, County Clinical Emergency Hospital of Bihor, 410169 Oradea, Romania; paliroxana@yahoo.com (R.G.P.); floreadarius91@gmail.com (D.G.F.); 7 Department of Environmental Engineering, Faculty of Environmental Protection, University of Oradea, 410048 Oradea, Romania; ateusdea@uoradea.ro

**Keywords:** major depressive disorder, depression severity, mental health, vitamin D, 25-hydroxyvitamin D, Romanian adults

## Abstract

The implication of vitamin D in the pathophysiology of depression has generated interest regarding its role as a biomarker in the context of mental health. Given the limited data on this association in the Romanian population, the present study aimed to evaluate the correlation between vitamin D status and the severity of depressive symptomatology in a sample of adult patients from Romania. This cross-sectional study was conducted on 90 patients diagnosed with major depressive disorder who were admitted to the Department of Psychiatry at the County Clinical Emergency Hospital of Bihor during the period of 2024–2025. Serum vitamin D concentrations were determined by measuring the 25-hydroxyvitamin D metabolite in blood using the chemiluminescence microparticle immunoassay method. The severity of depressive symptomatology was quantified using two validated scales: the Beck Depression Inventory-II (BDI-II) and the Hamilton Depression Rating Scale 17 (HAM-D17). Multivariable linear regression and ordered probit regression were used to assess the relationship between vitamin D status and depression severity, with adjustments for relevant covariates. A statistically significant negative association was identified for the BDI-II scale (B = −0.3170444, β = −0.2621328, *p* = 0.007) and the HAM-D17 scale (B = −0.1495026, β = −0.2829461, *p* = 0.002), which remained robust after adjustment for covariates (BDI-II: B = −0.2110473, β = −0.1744942, *p* = 0.050; HAMD17: B = −0.1153659, β = −0.2183397, *p* = 0.018). In comparison with subjects with vitamin D deficiency, those with sufficient levels presented a significantly lower probability of belonging to higher severity categories of depression for both scales (BDI-II: coefficient = −0.6855429, *p* = 0.004; HAM-D17: coefficient = −0.7029572, *p* = 0.005). The study highlighted a statistically significant negative correlation between vitamin D status and depression severity, which remained after adjustment for covariates. Sufficient vitamin D levels were associated with a significantly lower probability of belonging to a higher depression severity category compared with deficiency.

## 1. Introduction

Major depressive disorder (MDD) is a complex heterogenous neuropsychiatric disease that affects approximately 332 million people worldwide [1,2]. The estimated prevalence is 4% in general population with a rate of 5.7% among adults and with a higher percentage for women (6.9%) then men (4.6%) [1]. Commonly, it is characterized by a cluster of symptoms such as: concentration difficulties, thoughts associated with a lower self-esteem and feelings of guilt, pessimist vision for the future, suicide thoughts or death-related preoccupations, sleep disturbances, fatigue and lack of energy, body weight and appetite changes, and anhedonia [3].

From a clinical management perspective, the treatment of depression necessitates a multidisciplinary approach, including psychotherapy, pharmacotherapy, or a combination of both [4,5]. The actual limitations of conventional pharmacotherapy, manifested by suboptimal response rates and a higher incidence of adverse effects, have prompted a critical reevaluation of clinical protocols. Given the multidimensional impact of depressive disorder—amplified by socioeconomic stress factors from the SARS-CoV-2 pandemic period—it becomes imperative to integrate complementary therapeutic strategies aimed at improving patient adherence and optimizing long-term clinical outcomes [6]. Overall, the nutritional psychiatry literature suggests that dietary interventions are evaluated as potential adjuvant strategies for the prophylaxis and management of depression [7], and some evidence indicates that diet and associated dietary behaviors are indirectly correlated with the risk of depression, as well as with the severity and evolution of symptoms [8].

Vitamin D is a fat-soluble vitamin, considered both a neuroactive steroid [9] and a neurosteroid hormone [10]. It is naturally present in both plant- and animal-derived foods, fortified products, or synthesized endogenously [11] upon exposure to ultraviolet B (UVB) radiation on the skin [9]. Vitamin D is metabolized in the liver and kidneys, resulting in two metabolites: 25-hydroxyvitamin D (25(OH)D; calcidiol) and 1,25-dihydroxyvitamin D (1,25(OH)2D; calcitriol). 25(OH)D represents the principal circulating metabolite serving as the standard measure for assessing serum vitamin D concentration, while 1,25(OH)2D is the biologically active metabolite [9,12].

In recent years, the role of vitamin D as a relevant biomarker in the context of mental health has been the subject of growing scientific interest; however, the findings of existing studies remain inconsistent and, at times, contradictory. Some studies identified a negative association between vitamin D and depression [13,14,15,16,17], while others have failed to confirm the existence of such a link [18,19]. In the absence of original studies dedicated to investigating this relationship in adults diagnosed with MDD from Romania, the present study aimed to cross-sectionally evaluate the correlation between serum 25(OH)D concentration and the severity of depressive symptomatology, quantified using two standardized rating scales. Given the multifactorial nature of the underlying pathophysiological mechanisms, a secondary objective was to examine the influence of several relevant covariates—including medical history, sociodemographic characteristics, lifestyle, and environmental factors—on vitamin D levels, depression severity, and the correlation between these two parameters. Drawing on the data obtained from the existing literature, the following hypotheses have been formulated: (1) serum vitamin D levels are negatively associated with the severity of depressive symptoms, even after model adjustments for several covariates; and (2) the mentioned covariates can modulate vitamin D status, the clinical expression of depression, and the relationship between them. A deeper understanding of the role of vitamin D in mental health may offer valuable insights into the potential of nutritional interventions and lifestyle modifications in the management of affective disorders.

## 2. Results and Discussion

A total of 226 patients were assessed for eligibility between March 2024 and November 2025. After the exclusion of 136 subjects, the final analysis was conducted on a cohort of 90 patients. Reasons for non-participation: lack of interest in participating and refusal to sign the informed consent form (N = 92), inability to complete the questionnaires (N = 5), exclusion criteria (N = 18), declined blood sample collection (N = 10), and incomplete data (N = 11). The exclusion criteria were: pregnant and breastfeeding women, individuals with harmful use of alcohol or other substances, and patients with significant cognitive impairment that could interfere with the accurate completion of psychometric assessments.

**Figure 1:**
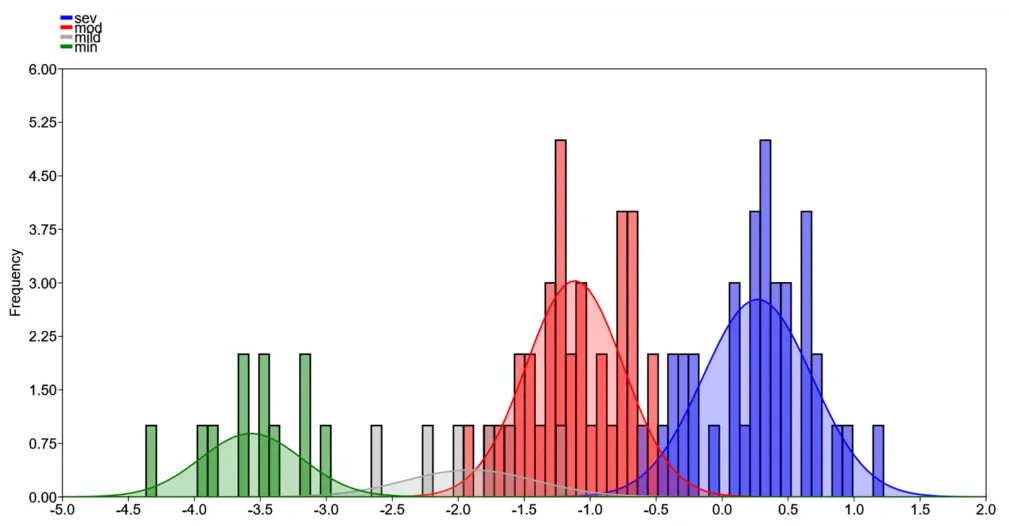
Distribution of raw BDI-II scores.

**Figure 2:**
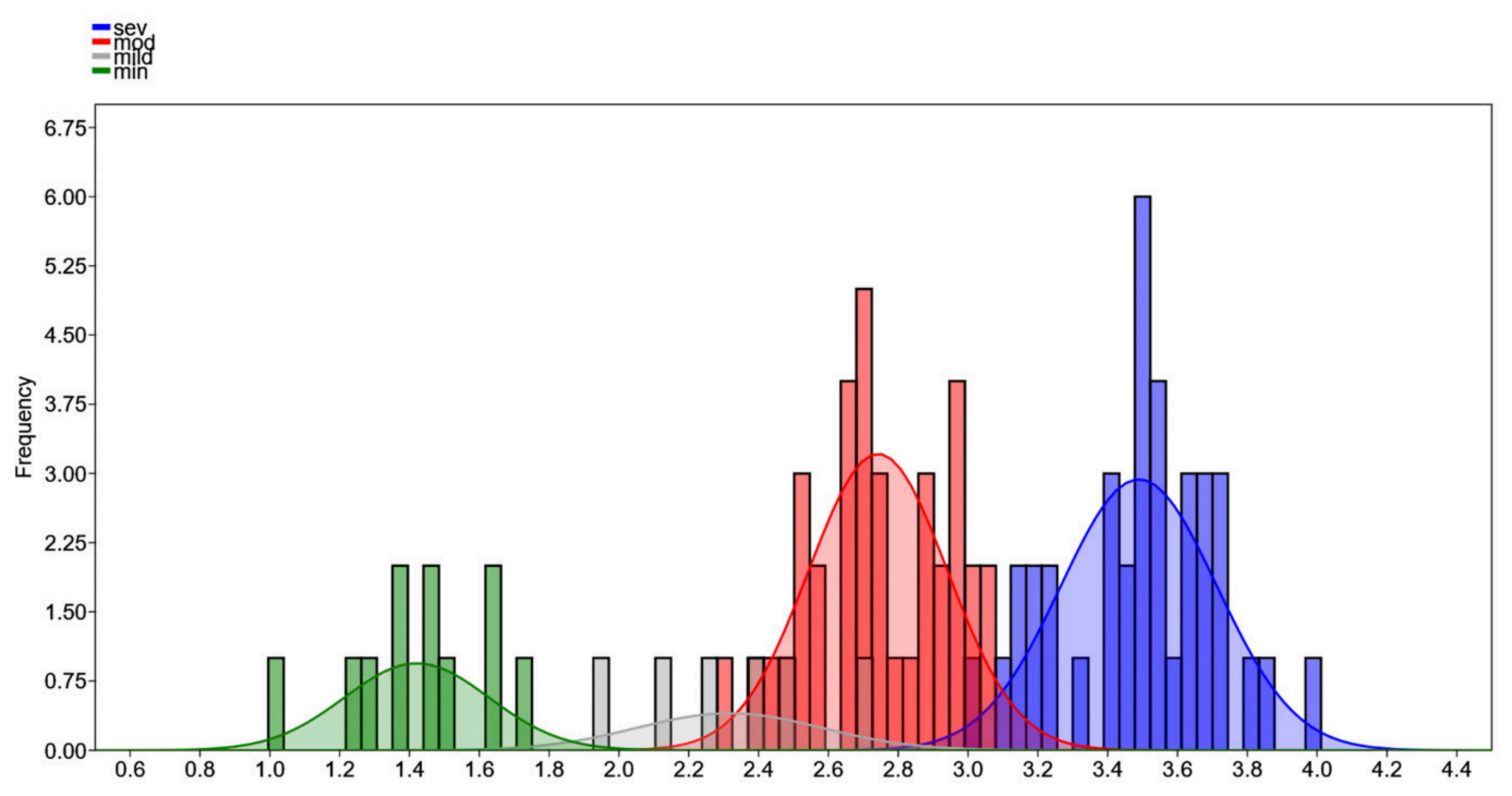
Distribution of normalized BDI-II scores.

**Figure 3:**
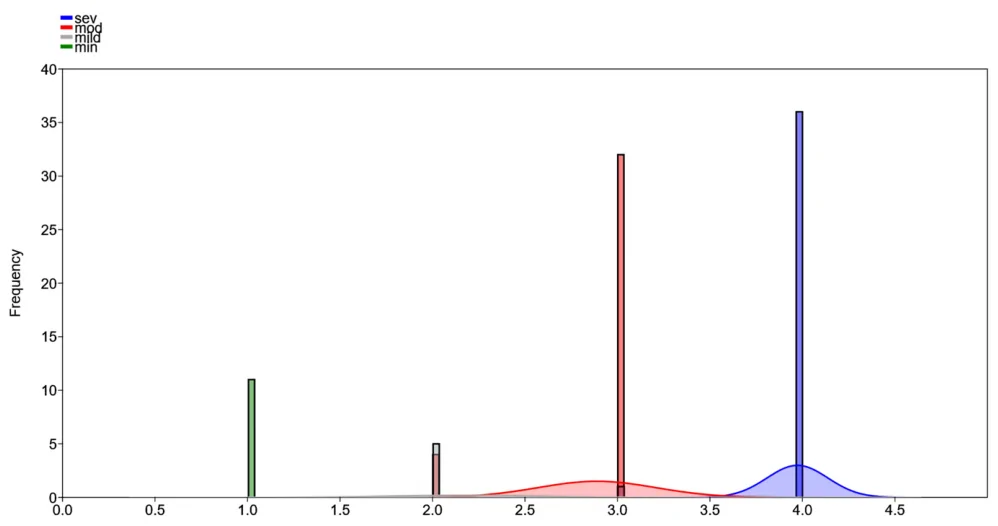
Distribution of final clinical severity categories of the BDI-II scale.

**Figure 4:**
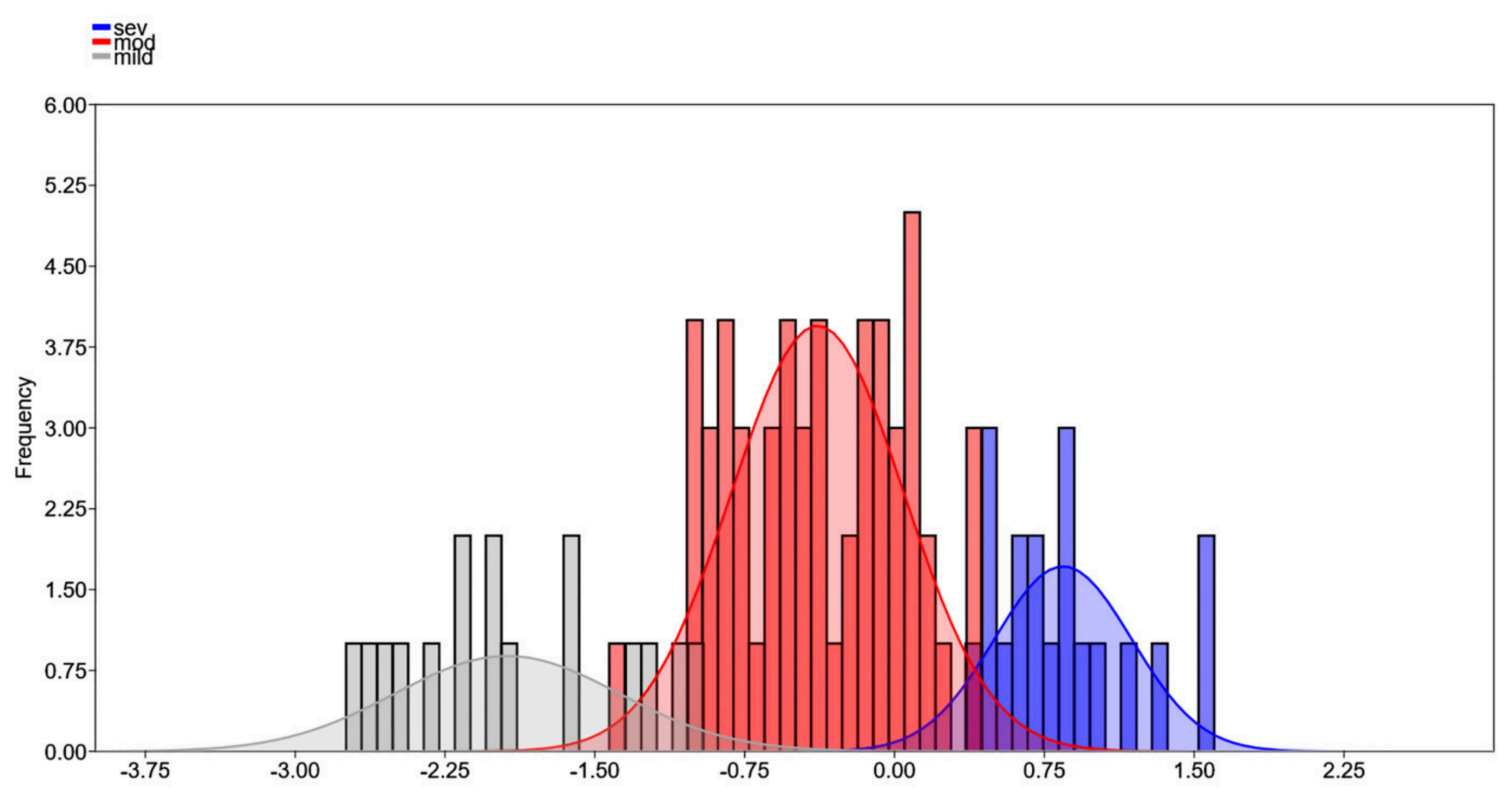
Distribution of raw HAM-D17 scores.

**Figure 5:**
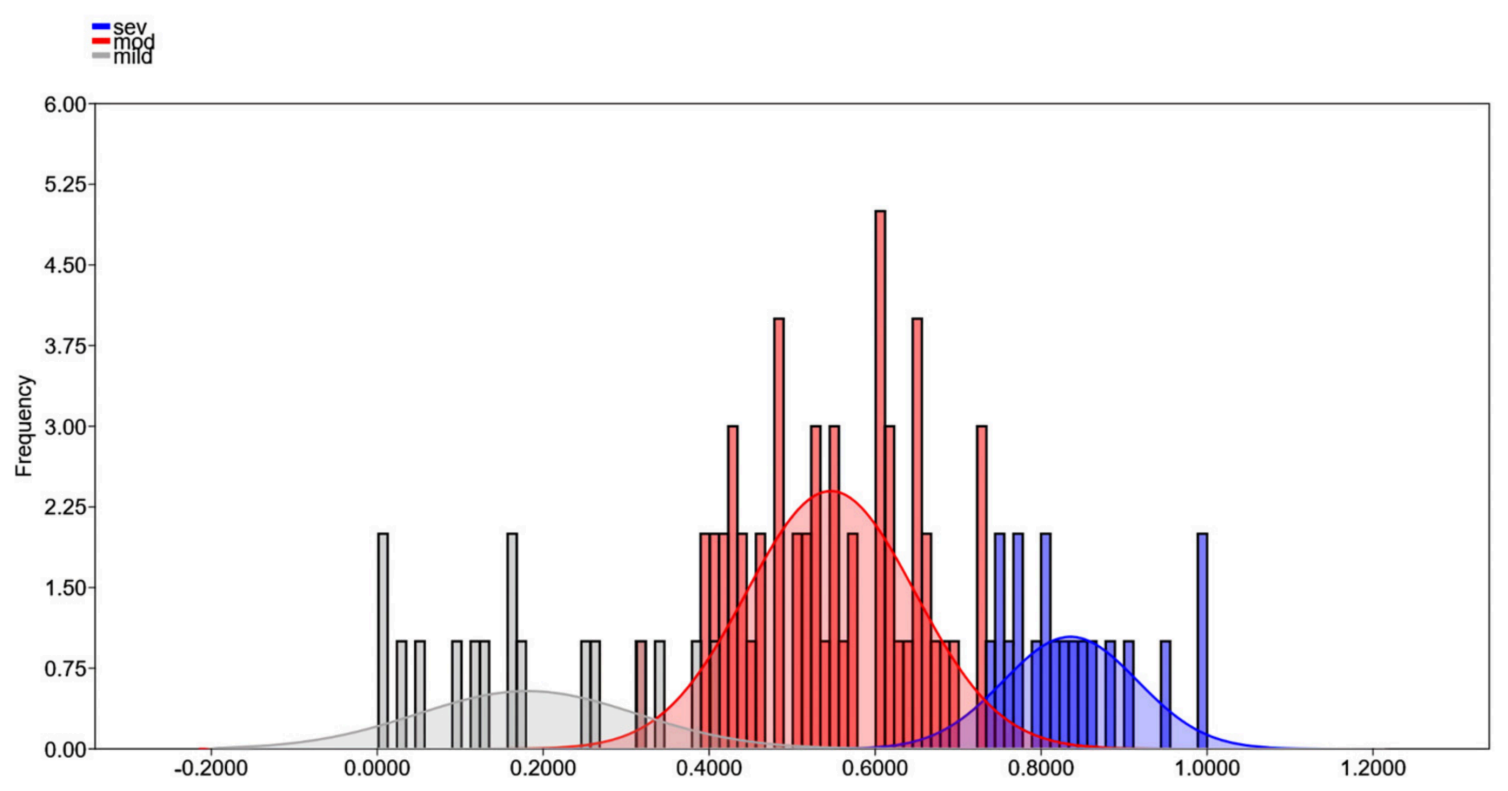
Distribution of normalized HAM-D17 scores.

**Figure 6:**
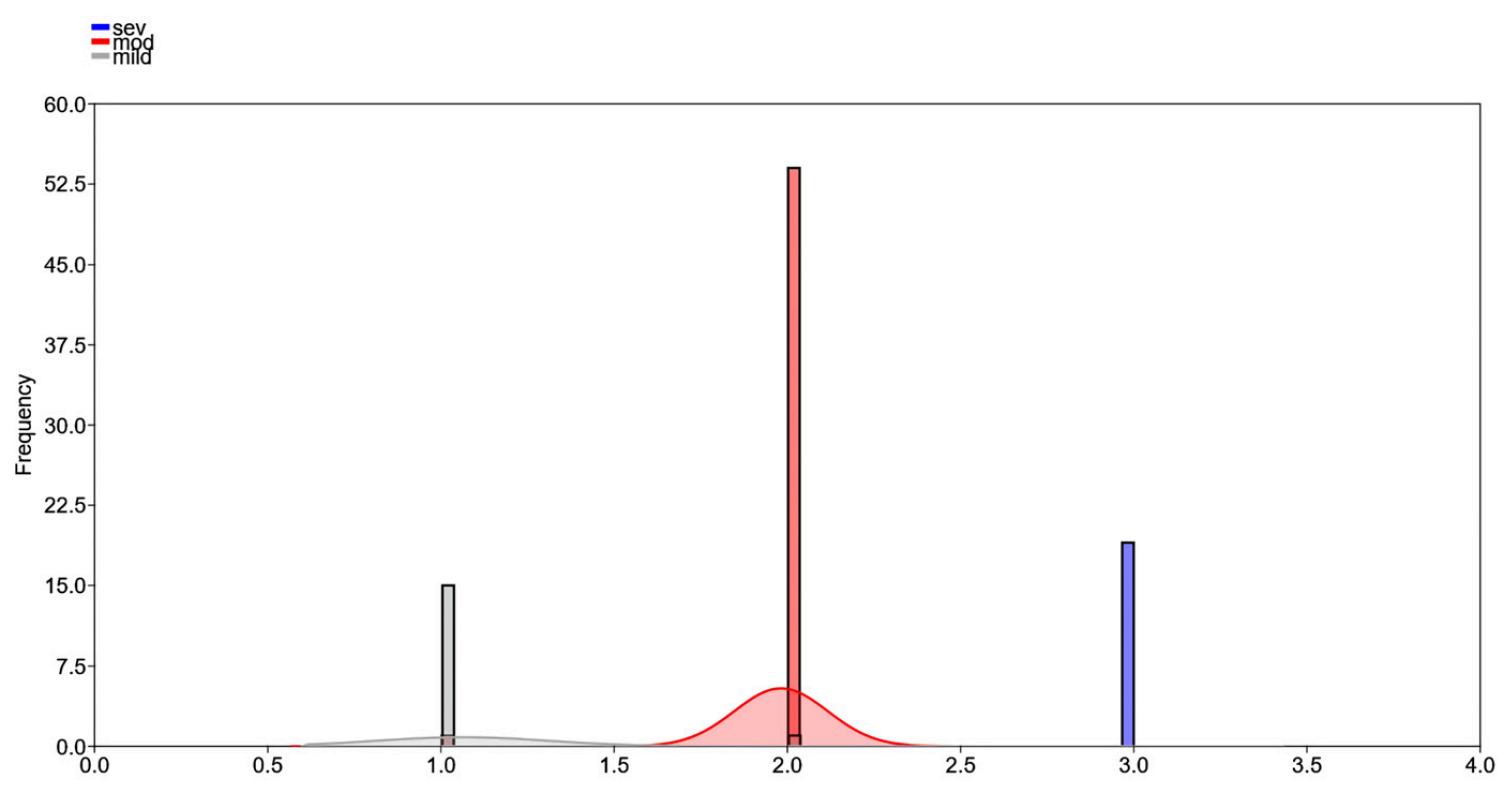
Distribution of final clinical severity categories of the HAM-D17 scale.

The descriptive analysis, which contains the distribution of sociodemographic, clinical, and biological characteristics of the investigated study group, is presented in Table 1. The linear regression models, which highlight the relationship between serum 25(OH)D concentrations (as the independent variable) and the BDI-II and the HAM-D17 depression scale scores (as dependent variables), are reported in Table 2. The ordered probit regression, specific to each of the two depression severity rating scales, is shown in Table 3 and Table 4. Table 5 and Table 6 are the classification tables, which present the observed and predicted severity categories of the ordered probit regression model for each rating scale. The distribution of raw predicted values, normalized values, and final clinical categories is illustrated in Figure 1, Figure 2, Figure 3, Figure 4, Figure 5 and Figure 6.

### 2.1. Descriptive Analysis of Sociodemographic, Clinical, and Biological Characteristics

A total of 90 participants diagnosed with MDD were included in the final analysis. The distribution of sociodemographic, clinical, and biological characteristics of the investigated group is presented in Table 1. With a non-normal distribution, the median age covariate was 56.00 (IQR = 8; 34–77), and the majority were female (N = 85, 94.44%). Most participants resided in rural areas (N = 52, 57.78%) and had medium-to-high educational attainment (N = 77, 85.56%). The majority were enrolled during the summer season (N = 64, 71.11%) and reported a sun exposure exceeding 30 min/day (N = 51, 56.67%). Most participants were overweight (N = 33, 36.67%) and exhibited a complex clinical profile characterized by two or more comorbidities (N = 49, 54.44%). The mean serum 25(OH)D concentration across the entire study population was 22.77 ± 9.058 ng/mL. Notably, 81.11% of the sample had 25(OH)D concentrations below the sufficiency threshold (<30 ng/mL), reflecting a high prevalence of vitamin D deficiency or insufficiency in this population. The mean HAM-D17 score was 20.50 ± 4.786, and the median BDI-II score was 25 (IQR = 13.75; 5–63). Most participants were classified as having moderate depression based on the HAM-D17 scale (N = 55, 61.11%), while the majority scored in the severe range on the BDI-II (N = 37, 41.11%). The mean total serum calcium concentration was 9.04 ± 0.526 mg/dL and the median of the estimated average daily dietary intake of vitamin D was 140.28 IU/day (IQR = 154; 6.14–1610.29).

### 2.2. Multivariable Linear Regression Analysis: Association Between Serum 25(OH)D Concentrations and Depression Severity

To evaluate the impact of serum 25(OH)D concentration (used as a continuous variable) on the severity of depressive symptomatology, an unadjusted linear regression model was initially applied (Model 0). To verify the independence of this relationship from confounding factors, sequential adjustment models were constructed (Models 1–3). To ensure internal validation of the results, the linear regression analysis was applied redundantly for both assessment scales used to evaluate depressive symptomatology (Table 2).

With respect to the BDI-II scale, the unadjusted model results indicated a statistically significant inverse relationship between the two variables (B = −0.3170444, β = −0.2621328, *p* = 0.007). From a clinical perspective, the B coefficient showed that, for each one-unit increase in vitamin D concentration (1 ng/mL), the depression score decreases by an average of 0.31 points. In Model 1, after statistical adjustment for baseline covariates (age and season of blood withdrawal), the direction of correlation remained (B = −0.3176379, β = −0.2626235, *p* = 0.006). Through the inclusion of lifestyle-related covariates in Model 2 (place of residence, education, BMI, the estimated average daily dietary intake of vitamin D, and sun exposure), a reduction in the strength of the association through both B and β coefficients was observed. Although an increase in the *p*-value was observed compared with the previous models, the correlation between vitamin D and depression score remained statistically significant (B = −0.2387353, β = −0.1973867, *p* = 0.025). Regarding Model 3, which was additionally adjusted for clinical covariates (total serum calcium and the number of chronic diseases), an attenuation of the absolute coefficient values (B = −0.2110473, β = −0.1744942) and an elevation in the *p*-value up to the threshold of statistical significance (*p* = 0.050) was recorded compared with Model 2.

The trend noted for the BDI-II scale was confirmed in the analysis of scores obtained using the HAM-D17 scale. Model 0 results revealed the presence of a negative relationship between serum 25(OH)D concentration and the severity of depressive symptomatology. In contrast to the BDI-II scale, for the HAM-D17 scale, the *p*-value and both B and β coefficients were lower, particularly the B coefficient (B = −0.1495026, β = −0.2829461, *p* = 0.002). For Model 1, the significance of the association persisted, with values comparable to those of Model 0 (B = −0.146535, β = −0.2773298, *p* = 0.003). The Model 2 analysis showed an attenuation in the strength of the correlation (B = −0.1222592, β = −0.2313858) and an increase in the *p*-value to 0.010. For Model 3, adjusted for clinical factors, a reduction in the absolute coefficient values (B = −0.1153659, β = −0.2183397) was highlighted. Although the *p*-value increased compared with the previous model, it remained well below the threshold of statistical significance (*p* = 0.018).

### 2.3. Ordered Probit Regression: The Probability of Membership in the Clinical Depression Severity Categories

Given that the multivariable linear regression analysis confirmed the existence of a significant association between vitamin D status and the severity of the depressive episode, ordered probit regression was additionally applied separately for each assessment scale—the BDI-II and the HAM-D17 (Table 3 and Table 4). The observed and predicted severity categories of the ordered probit regression model for the BDI-II and HAM-D17 scales are presented in Table 5 and Table 6. The distribution of raw predicted values, normalized values, and final clinical categories is illustrated in Figure 1, Figure 2, Figure 3, Figure 4, Figure 5 and Figure 6.

For the BDI-II scale, the ordered probit regression model achieved global statistical significance (Wald chi^2^ (14) = 24.26, Prob > chi^2^ = 0.0427). Compared with the reference category—vitamin D deficiency—patients with sufficient vitamin D levels presented a significantly lower probability of belonging to a higher depression severity category (coefficient = −0.6855429, *p* = 0.004). The association between insufficient vitamin D levels and the severity of depression had the same direction; however, it failed to reach statistical significance (coefficient = −0.2868912, *p* = 0.139). Regarding age categories, using the 30–49-year age group as a reference, subjects from the 50–59-year age group presented a greater probability of belonging to a higher depression severity category (coefficient = 0.7329627, *p* = 0.013). Among participants aged over 60 years, the probability of belonging to a higher depression severity category was lower, albeit statistically non-significant (coefficient = −0.0009885, *p* = 0.997). Total serum calcium and medium-to-high educational level—the reference being lower educational level—were significantly and negatively associated with the severity of depression (total serum calcium: coefficient = −0.3741122, *p* = 0.036; educational level: coefficient = −0.6392058, *p* = 0.040). The remaining covariates included in the model failed to reach statistical significance (*p* ≥ 0.05). The cut-points estimated by the model were: cut1 = −3.084909, cut2 = −2.360221, cut3 = −0.3766894. The classification table indicated a 93.33% global accuracy of the model, with 84 out of 90 participants correctly classified. Accuracy per category was: 100.00% for minimal category, 83.33% for mild category, 88.89% for moderate category, and 97.30% for severe category.

**Table 4:** Ordered probit regression analysis of depression severity assessed by HAM-D17.

numHAM-D17	Coefficient	Robust Std. Err.	*p*-Value
numAGE			
50–59	0.8162553	0.3384619	0.016
60+	0.2688533	0.3292805	0.414
num25OHD			
insuf	0.1743533	0.2247649	0.438
suf	−0.7029572	0.24751	0.005
numISE			
15–30	−0.2505208	0.3267619	0.443
>30	−0.2921519	0.2319697	0.208
numSOBW			
Winter.Time	0.24825	0.2129351	0.244
numRSD			
U	0.1942905	0.2127062	0.361
numOCD			
1	0.0670319	0.2875476	0.816
≥2	0.125237	0.2794992	0.654
numED			
MED-HIGH	−0.4768346	0.2933488	0.104
tsca	−0.2657131	0.2053298	0.196
bmi	0.0006564	0.0193293	0.973
dvdi	−0.0005156	0.0005776	0.372
numHAM-D17	1	(offset)	
/cut1	−1.775446	1.966276	
/cut2	0.8016538	1.930237	

Reference categories: numAGE—30–49; num25(OH)D—deficiency; numISE—<15; numSOBW—Summer.Time; RSD—rural; numOCD—0; numED—low. Abbreviations: 25(OH)D = 25-hydroxyvitamin D; BMI = body mass index; cut1, cut2 = cut-points estimated by maximum likelihood; dvdi = the estimated average daily dietary intake of vitamin D; ED = education; insuf = insufficiency; HAM-D17 = Hamilton Depression Rating Scale 17; ISE = sun exposure; MED-HIGH = medium-to-high; OCD = number of chronic diseases; RSD = place of residence; SOBW = season of blood withdrawal; suf = sufficiency; std.err. = standard error; tsca = total serum calcium; U = urban.

The ordered probit regression model for the HAM-D17 scale achieved global statistical significance (Wald chi^2^ (14) = 30.45, Prob > chi^2^ = 0.0066), demonstrating superior global significance compared with the BDI-II scale. Compared with the reference category—vitamin D deficiency—the patients with sufficient vitamin D levels presented a significantly lower probability of belonging to a higher depression severity category (coefficient = −0.7029572, *p* = 0.005), a result consistent with that of the BDI-II scale. However, in contrast to the BDI-II scale, where both vitamin D categories had negative coefficients compared with the reference category, for the HAM-D17 scale, the insufficient group yielded a positive coefficient that was statistically non-significant (coefficient = 0.1743533, *p* = 0.438). Using the same reference for the age covariate, the subjects from the 50–59-year age group demonstrated a greater probability of belonging to a higher depression severity category (coefficient = 0.8162553, *p* = 0.016), a pattern also observed for the BDI-II scale. For the HAM-D17 scale, a difference emerged regarding the individuals aged over 60 years, who were positively but statistically non-significantly associated with the severity of depression (coefficient = 0.2688533, *p* = 0.414). The remaining covariates included in the model failed to reach statistical significance (*p* ≥ 0.05). The cut-points estimated by the HAM-D17 scale model were: cut1 = −1.775446, cut2 = 0.8016538. In contrast to the BDI-II scale, the HAM-D17 classification table indicated a 97.77% superior global accuracy with 88 out of 90 participants correctly classified. Accuracy per category was: 93.75% for the mild category, 98.18% for the moderate category, and 100.00% for the severe category.

**Table 5:** Classification table—observed vs. predicted severity categories for BDI-II.

numBDI-II	pred_BDI-II (Linear Prediction (Cut-Points Excluded))				Total
	pred_min	pred_mild	pred_mod	pred_sev	
min	11	0	0	0	11
	100.00	0.00	0.00	0.00	100.00
	100.00	0.00	0.00	0.00	12.22
mild	0	5	1	0	6
	0.00	83.33	16.67	0.00	100.00
	0.00	55.56	2.94	0.00	6.67
mod	0	4	32	0	36
	0.00	11.11	88.89	0.00	100.00
	0.00	44.44	94.12	0.00	40.00
sev	0	0	1	36	37
	0.00	0.00	2.70	97.30	100.00
	0.00	0.00	2.94	100.00	41.11
Total	11	9	34	36	90
	12.22	10.00	37.78	40.00	100.00
	100.00	100.00	100.00	100.00	100.00
Pearson chi^2^ (9) =		209.4855		Pr = 0.000	
Likelihood-ratio chi^2^ (9) =		180.1389		Pr = 0.000	
Cramér’s V =		0.8808			
gamma =		0.9969		ASE = 0.003	
Kendall’s tau-b =		0.9498		ASE = 0.021	
Fisher’s exact =				0.000	

Key: Data are presented as absolute frequency (N), row percentage (%), and column percentage (%). Abbreviations: BDI-II = Beck Depression Inventory-II; min = minim; mod = moderate; pred = predicted; sev = severe.

**Table 6:** Classification table—observed vs. predicted severity categories for HAM-D17.

numHAM-D17	pred_HAM-D17 (Linear Prediction (Cut-Points Excluded))			**Total**
	pred_mild	pred_mod	pred_sev	
mild	15	1	0	16
	93.75	6.25	0.00	100.00
	93.75	1.82	0.00	17.78
mod	1	54	0	55
	1.82	98.18	0.00	100.00
	6.25	98.18	0.00	61.11
sev	0	0	19	19
	0.00	0.00	100.00	100.00
	0.00	0.00	100.00	21.11
Total	16	55	19	90
	17.78	61.11	21.11	100.00
	100.00	100.00	100.00	100.00
Pearson chi^2^ (4) =.		166.0631	Pr = 0.000	
Likelihood-ratio chi^2^ (4) =		151.0699	Pr = 0.000	
Cramér’s V =		0.9605		
gamma =		0.9991	ASE = 0.001	
Kendall’s tau-b =		0.9681	ASE = 0.023	
Fisher’s exact =			0.000	

Key: Data are presented as absolute frequency (N), row percentage (%), and column percentage (%). Abbreviations: HAM-D17 = Hamilton Depression Rating Scale 17; min = minim; mod = moderate; pred = predicted; sev = severe.

The present cross-sectional study investigated the relationship between serum 25(OH)D concentration and the severity of depressive symptomatology in a sample of Romanian adults diagnosed with MDD. The statistical analyses, independently performed for the two validated depression rating scales (the BDI-II and the HAM-D17), indicated a significant negative correlation between vitamin D status and the severity of depression scores. This link remained robust even after adjusting the regression models for sociodemographic, clinical, and biological covariates.

In terms of the BDI-II depression scale, the statistical analysis revealed the robustness of the association between vitamin D and affective symptoms. The quasi-absolute stability of B and β coefficients in Model 1 demonstrated that the investigated correlation was completely independent of baseline covariates (age and season of blood withdrawal). Subsequently, although the introduction of lifestyle-related covariates in Model 2 led to an increase in the *p*-value and a modest reduction in the absolute values of B and β coefficients, the maintenance of statistical significance below the 0.05 threshold supports an independent association of 25(OH)D on depression severity. This pattern suggests that, while factors such as place of residence, education, BMI, the estimated average daily dietary intake of vitamin D, and sun exposure may account for a portion of the variance in depression scores, serum vitamin D concentration maintains its own independent and significant association with depression severity. The inclusion of clinical covariates in Model 3 resulted in a marginal attenuation of the association of only 0.02, even as the *p*-value reached 0.050. This trend suggests that, even though the fully adjusted model becomes more conservative due to a reduction in statistical power, the primary clinical signal remains stable, supporting hypovitaminosis D as an independent correlate of depression severity. The reproducibility of the linear regression analysis for the HAM-D17 scale confirmed the robustness of the clinical signal in the study population, even though notable differences were observed between the two assessment tools. While Model 0 presented a lower value for the B coefficient, the correlation was highly statistically significant, similar to the BDI-II scale, remaining fully unaffected by baseline covariates from Model 1. The introduction of lifestyle-related covariates in Model 2 reproduced the pattern of coefficient attenuation, reconfirming the modulatory role of behavioral habits in this relationship. The major divergence appears for Model 3, whereas for the BDI-II scale, the fully adjusted model drove the *p*-value toward the statistically significant threshold; for the HAM-D17 scale, the *p*-value remained stable well within the significance threshold. This statistical discrepancy indicates that these two rating scales capture different symptomatology dimensions, reflecting the longstanding debate in the scientific literature regarding the psychometric instrument convergence. While the BDI-II scale is a self-report instrument grounded in Beck’s cognitive theory, focused on capturing patient’s subjective perceptions and cognitive distortions [20], the HAM-D17 scale is a clinician-administered rating scale that objectively quantifies somatic and behavioral manifestations [21]. This conceptual delimitation explains why, in the study, the fully adjusted model exhibited a different response for the two scales. The HAM-D17 scale identified a robust independent somatic component closely linked to the biological parameters (such as vitamin D status) even after the adjustment for the number of chronic diseases and total serum calcium, while the BDI-II scale appeared more susceptible to subjective interference arising from comorbidities and serum calcium variations.

The results obtained in linear regression analysis were confirmed and refined through ordered probit regression analysis, which allowed for the estimation of the probability of membership in the clinical depression severity categories based on vitamin D status and the covariates included in the model. While both models achieved global statistical significance, the HAM-D17 scale model demonstrated superior global significance (Wald chi^2^ (14) = 30.45, Prob > chi^2^ = 0.0066) compared with the BDI-II scale model (Wald chi^2^ (14) = 24.26, Prob > chi^2^ = 0.0427). For both rating scales, the sufficient vitamin D category was significantly and negatively associated with the probability of belonging to a higher depression severity category compared with the reference category—vitamin D deficiency—thus confirming the linear regression analysis results. Regarding the insufficient vitamin D category, a statistically non-significant result was obtained for both scales, a finding that may suggest that the relevant clinical difference occurs at the threshold between deficiency and sufficiency. Notably, in contrast to the BDI-II scale, where the insufficient vitamin D category had a negative coefficient, for the HAM-D17 scale, the corresponding coefficient was positive. This finding further reflects the differences between these two scales in terms of capturing the depressive symptoms. With respect to the classification tables related to the predictive analysis, the global accuracy of the HAM-D17 scale model (97.77%) was superior to the BDI-II scale model (93.33%), thus reflecting the greater capacity of objective clinical assessment to capture the neurobiological severity of depression in relationship with vitamin D status. Both models had adjacent classification errors, with the lack of major inconsistencies between extreme severity categories lending acceptable clinical relevance to the findings.

The association identified in the study confirms the pattern reported in the scientific literature. Hollinshead et al. conducted a comprehensive analysis including 11,337 participants (pregnant women, postpartum women, non-pregnant/postpartum women, and men), aged between 20 and 44 years. Using the Patient Health Questionnaire-9 (PHQ-9) scale, the authors showed an inverse significant relationship between the severity of depressive symptomatology and serum vitamin D concentrations [15]. This trend is corroborated by Zhu et al., who examined 50 patients diagnosed with MDD. The results indicated that depressive symptoms, quantified by the HAMD scale, were more severe in patients with lower 25(OH)D concentrations [14]. Furthermore, a sample of 3369 middle-aged and older men (aged between 40 and 79 years) participating in the European Male Aging Study (EMAS), was followed up to investigate the association between serum vitamin D concentration and depression. The univariate analysis revealed significantly decreased levels of 25(OH)D in persons with depressive symptoms (*p* < 0.001). The evaluation conducted through the BDI-II scale confirmed the negative relationship between the two variables, the association remaining statistically significant even after adjustment for multiple covariates [13]. These studies, through their complexity and relevance, share essential methodological and clinical convergence with the present study.

Two meta-analyses demonstrated, on extensive samples, that vitamin D deficiency represents a significant risk factor for the onset and severity of depressive symptomatology [22,23]. Anglin et al. conducted a systematic review and meta-analysis to investigate the correlation between vitamin D deficiency and depression in adults. One case–control study, ten cross-sectional studies, and three cohort studies, comprising a total of 31,424 participants, were analyzed. Low vitamin D levels were identified in persons with depression compared with the control group. Furthermore, in the cross-sectional studies, an increased odds ratio for the occurrence of depression was observed in the categories with the lowest vitamin D levels in contrast to those with the highest levels [22]. A recent umbrella meta-analysis, which synthesized evidence from multiple international databases till March 2022, offered a comprehensive overview of this matter. The analysis of four meta-analysis of cohort studies demonstrated that low serum vitamin D levels were associated with significantly greater odds of depression compared with high levels. This high-level synthesis confirms not only the existence of a statistical association, but also the potential benefits of maintaining elevated serum 25(OH)D concentrations in reducing the risk of occurrence and the severity of depressive symptoms [23], results that converge with the identified findings from our study population.

Nevertheless, the scientific literature lacks unanimity, as some studies have not reported an association between serum vitamin D concentration and depression. For instance, Mousa et al. investigated a sample of overweight or obese adults with vitamin D deficiency. The results failed to demonstrate a statistically significant correlation between vitamin D status and the severity of depressive symptoms assessed using the BDI scale [18]. Likewise, a study conducted on the Danish general adult population reported that low serum 25(OH)D levels were not associated with mean depression and anxiety scores in quantile median regression analysis adjusted for sex, age, and other potential confounders [19]. These conflicting findings reported may result from study design heterogeneity, sample size, analytical approaches used, and sociodemographic characteristics of the participants. The methodological quality of the studies, the application of different diagnostic criteria for depression, the use of self-report assessment tools instead of clinical interviews, the variability of laboratory methods used to measure the serum 25(OH)D concentration, and the presence of uncontrolled residual confounders may explain the discrepancies identified in the literature [23,24,25].

The association observed in this cross-sectional analysis is consistent with data obtained from longitudinal and randomized clinical trials. A longitudinal study demonstrated that hypovitaminosis D represents a significant predictive factor for mental health, being associated with a 75% higher risk of developing depression over a four-year period [26]. Similarly, randomized clinical trials, such as that conducted by Alghamdi et al., confirmed that vitamin D supplementation may improve symptom severity in depression, an effect particularly observed in women [27]. These findings strengthen the validity and clinical relevance of our results.

The pathophysiology of MDD is complex and not yet fully elucidated, with multiple convergent neurobiological mechanisms being described, such as alterations in monoaminergic neurotransmission, neuroendocrine changes, impairment of neurogenesis, redox and bioenergetic imbalances, and cytokine dysregulation consistent with a chronic inflammatory profile [28]. Within the monoaminergic hypothesis, dysregulation of serotonin, dopamine, and norepinephrine is considered relevant to the emergence of depressive symptoms [29]. Antidepressants act by increasing the synaptic availability of these neurotransmitters by blocking the reuptake of serotonin and norepinephrine at presynaptic nerve terminals [30]. Elevated levels of proinflammatory cytokines were associated with disruption of the hypothalamic–pituitary–adrenal (HPA) axis, alteration in glutamatergic neurotransmission, reduction in neuroplasticity, dysbiosis of the gut microbiota, and disturbances in tryptophan (TRP) metabolism [31]. Regarding its etiology, this is likely multifactorial, encompassing both non-modifiable and modifiable factors such as genetic predisposition, environmental factors (life events, limited social support, and financial difficulties), and lifestyle behaviors, including diet, whose role in mental health is increasingly recognized [5].

The presence of vitamin D metabolites in the cerebrospinal fluid of healthy adults has generated growing interest in the potential involvement of this vitamin in neurodevelopment and cerebral function processes [32]. From a biological perspective, this hypothesis seems plausible since vitamin D is capable of crossing the blood–brain barrier, ultimately reaching the central nervous system (CNS) [33], where it may activate specific receptors in different brain regions, including the hippocampus and prefrontal cortex—areas associated with the regulation of mood, affect, and emotional response [34]. Beyond the expression of specific receptors, the scientific literature also describes the presence of the enzyme required for active metabolite synthesis [35]. Even though the vitamin D mechanism in the brain is not fully elucidated [36], growing evidence indicates that it may have a significant impact on brain development and function through the maintenance of calcium homeostasis and signaling, regulation of neurotrophic factors, neuroprotection, modulation of neurotransmission, and sustaining synaptic plasticity [37].

Although numerous studies have reported an inverse association between serum vitamin D concentration and the severity of depressive symptomatology [13,14,15,16,17], the direction of causality remains unclear; vitamin D deficiency potentially represents a risk factor, but also a cause of depression [16,38,39]. The absence of depressive symptomatology in some patients with deficiency, and, conversely, the presence of the disorder in persons with optimal levels, suggests the existence of complex multifactorial pathophysiological mechanisms [9]. As further suggested by the present study’s findings, a relatively higher number of covariates, such as age, season of blood withdrawal, education, place of residence, the estimated average daily dietary intake of vitamin D, BMI, sun exposure, the number of chronic diseases, and total serum calcium, may influence the severity of depression, serum 25(OH)D concentration, as well as the link between these two parameters.

The clinical relevance of the association between vitamin D deficiency and depressive pathology may be particularly important for older adults, a group in which lower circulating levels of 25(OH)D are identified with a higher frequency [40,41]. This biological status, specific to the advanced stages of life, may be explained through numerous mechanisms. Thus, the decrease in skin capacity to synthesize vitamin D precursors, correlated with reduced exposure to UV radiation due to limited mobility or living in care centers, and along with frequently inadequate dietary intake, represents major barriers to maintaining an optimal level. In addition, the presence of chronic diseases, changes in adipose tissue composition, and the progressive decline in renal function further contribute to the alteration in vitamin D metabolism [42,43,44]. However, conflicting results have been reported by Alharbi et al., who identified lower vitamin D levels among younger individuals compared with older adults [45]. This discrepancy reflects the heterogeneity of findings in the scientific literature and suggests that the relationship between age and vitamin D status does not follow a uniform pattern. Regarding the study results, the absence of a linear pattern was confirmed, as the lowest mean 25(OH)D levels were recorded in the over-60-year age group, followed by the 30–49-year age group, while the 50–59-year age group presented the highest mean value.

From the sociodemographic category, the educational level exerts a significant influence on the association between vitamin D status and mental health. Our findings are consistent with the scientific literature confirming that limited educational attainment is associated with a greater probability of reporting depressive symptoms, often coexisting with reduced serum 25(OH)D levels. A study conducted by Kwon et al. in a sample of women working in the manufacturing industry concluded that the level of education was significantly associated with depressive symptomatology, particularly among those with medium or lower education (high school diploma or below). The correlation between hypovitaminosis D and depressive symptoms maintains its statistically significance even after rigorous control of confounding variables, such as age, marital status, shift work schedule, and educational level [46].

The study results highlighted a nonlinear pattern of serum vitamin D concentrations according to BMI categories, with the lowest mean value recorded in the underweight group and maximum values in the overweight category, followed by a modest decline in the obesity categories. Even though the Spearman correlation coefficient indicated a negative association between serum vitamin D concentrations and BMI, this failed to reach statistical significance (*p* = 0.863). The observed vitamin D deficiency in the underweight group may be explained by insufficient caloric and lipid intake characteristic of this category, given that vitamin D has superior intestinal absorption in the presence of dietary fats [47]. The progressive increase in mean vitamin D values toward the overweight category may reflect a more substantial dietary intake, without adiposity having reached the threshold at which sequestration mechanisms become dominant. This observation is in concordance with the study of Alharbi et al., who reported greater levels of 25(OH)D in overweight subjects compared with normal-weight individuals and the absence of a relationship between these two parameters. The modest decrease in serum concentrations in the obesity categories is in accord with the mechanisms described by the literature. The primary proposed explanations include the sequestration of vitamin D in adipose tissue and volumetric dilution, through which vitamin D is distributed across a larger volume of adipose, muscle, and hepatic tissues, which reduces its circulating serum concentrations [45]. Another mechanism may involve the impairment of the hepatic hydroxylation process secondary to non-alcoholic fatty liver disease (NAFLD) [48]. Nevertheless, in the study population, the decrease among the obesity categories remained modest, with values hovering around the insufficiency threshold.

Vitamin D status is influenced by a complex multifactorial interaction involving the intensity and duration of sun exposure [49], characteristic seasonal variations [50], the geographical particularities of the residential area, and the impact of atmospheric pollution [51]. Sun exposure represents an essential confounding factor in the analysis of the link between vitamin D and depression [49]. From a physiological perspective, the endogenous production of cholecalciferol is initiated in the epidermis, where 7-dehydrocholesterol (7-DHC) absorbs the energy of UVB photons (290–315 nm), is cleaved, and is converted into previtamin D3. The efficiency of this process depends on numerous factors that may limit cutaneous synthesis, such as the use of sunscreen products, skin pigmentation, aging process, geographical latitude, season, and the timing of sun exposure [52]. In the context of mood disorders, a reverse causality appears whereby persons with depressive symptomatology tend to adopt sedentary behaviors and spend more time indoors, thus reducing opportunities for exposure to UVB radiation necessary for synthesis, which predisposes them to the onset and worsening of vitamin D deficiency [53]. The season of blood withdrawal represents another relevant confounding variable, as the time of year may exert a significant influence on vitamin D status and depressive symptomatology [54]. Regarding the seasonal variation in vitamin D levels, research has demonstrated that serum levels are, on average, 5 nmol/L higher during summer months compared with winter, a trend observed in both women and men [55]. A study conducted by Zheng et al. in patients diagnosed with bipolar depression showed a significant association between the season of hospitalization and vitamin D level profile; compared with a medium-level profile, patients admitted during autumn and winter presented a 3.32-fold higher risk of being classified in a low-level profile, while patients admitted in spring and summer recorded a 6.20-fold greater probability of being categorized in a high-level profile [56]. Concerning the impact of seasonality on depressive symptomatology, it is more pronounced in the context of seasonal affective disorder, whose prevalence is higher in the winter months [54], during which reduced UVB radiation exposure is considered a primary causative factor [57]. From the obtained data, it emerged that more prolonged exposure was associated with slightly elevated serum 25(OH)D levels and, simultaneously, with a modest attenuation of the severity of depressive symptomatology quantified through the HAM-D17 scale. Regarding the season of blood withdrawal, during summer months, the mean serum vitamin D concentration recorded a very slight but statistically non-significant increasing trend, while the mean HAM-D17 scores presented a moderate decrease.

A study that monitored the vitamin D status in postmenopausal women engaged in outdoor activities, either in Brussels or in rural areas, highlighted that hypovitaminosis D presented a stronger correlation with urban residence and air pollution, independent of a high sun exposure index. Compared with rural residents, urban dwellers recorded a greater sun exposure index; yet the prevalence of vitamin D deficiency (<75 nmol/L) was more than twice as high as that observed in rural areas (84% compared with 38%). This inverse correlation may be attributed to ozone concentrations three times higher in urban areas, which acts as a protective screen against the UVB radiation required for cutaneous synthesis, thereby diminishing vitamin D status despite time spent outdoors [51]. In the study sample, the findings indicated a similar trend; thus, the variation between the two means was minimal, with a slightly higher mean vitamin D value in rural compared with urban areas. The proximity of these values suggests that, although the direction of the association is consistent with the existing scientific literature, in the study group, the impact of residential environment is subtle. Hoogendijk et al. highlighted that urbanization level, albeit with a modest explanatory effect, confirms that lower sun exposure contributes to both vitamin D reduction and the onset of depression [58]. Interestingly, in the sample, while rural areas presented marginally elevated vitamin D levels, the prevalence of depressive symptoms remained higher in this area, with results that varied according to the rating scale used. The HAM-D17 scale indicated a negligible and statistically non-significant mean variation between the two places of residence, while the BDI-II scale showed a more statistically relevant discrepancy, with the median scores being higher in participants from rural areas.

Serum vitamin D concentration may be influenced by lifestyle changes, such as a reduction in physical activity and alterations in diet, which often emerge as a consequence of depression [59]. Negative mood may reduce appetite, generating changes in dietary habits that lead to insufficient dietary vitamin D intake [60]. As reported by Collin et al., this relationship appears to be mediated by the general dietary quality and the severity of the deficiency. Whereas a high-quality diet may reduce the psychological impact of moderately low 25(OH)D levels (<20 or <30 ng/mL), the risk of recurrent depressive symptoms remains higher for severe deficiency (<10 ng/mL), independent of dietary quality. This aspect suggests that a slightly insufficient 25(OH)D status may not adversely affect depressive symptoms when overall dietary quality is high, while a severe vitamin D deficiency may not be offset by a high-quality diet alone [61]. Regarding the obtained findings, the statistically non-significant relationship identified between the estimated average daily dietary intake of vitamin D and its serum concentration does not reflect a lack of dietary relevance, but rather a proportionally modest contribution relative to sun exposure, which presented a statistically significant impact. This hypothesis supports the important role of cutaneous synthesis in the modulation of serum 25(OH)D concentrations. The modest, but statistically significant relationship observed between HAM-D17 depression scores and the estimated average daily dietary intake of vitamin D suggests that the severity of clinically evaluated depressive symptoms may be associated with a decreased dietary intake, possibly reflecting dietary changes in the context of depression. The absence of a significant correlation for the BDI-II scale warrants caution in generalizing this interpretation.

The scientific literature highlights that hypovitaminosis D is common in patients with chronic diseases [23], a trend that is also consistent with the findings of the present study. The comorbidities may influence vitamin D status through multiple mechanisms, such as the reduction in outdoor activities, metabolic perturbations that may affect the conversion of vitamin D into its active form, the presence of chronic inflammation, or the use of medications that decrease the serum 25(OH)D levels [43]. The absorption efficiency of vitamin D from dietary sources and supplements depends on the integrity of the gastrointestinal system, which can be undermined by the presence of some pathologies, such as Crohn’s disease, celiac disease, lactose intolerance, or malabsorption and short bowel syndromes. Serum 25(OH)D concentration is critically determined by the state of liver and kidney function. Certain systemic disorders—hyperparathyroidism, granulomatous processes (sarcoidosis or tuberculosis), fungal infections such as histoplasmosis, and lymphatic cancer—may disrupt the metabolic balance of vitamin D [6]. Chronic conditions represent an important covariate capable of influencing serum vitamin D levels through sun exposure limitation, as well as the risk of depression [62]. The relationship between chronic pathology and mental health is bidirectional; while chronic conditions increase vulnerability to depression, depressive status may exacerbate the patient’s physical status [54]. The lowest mean serum 25(OH)D concentration was recorded in patients with two or more comorbidities. The mean values analysis indicated a mild increasing trend in the severity of depressive symptomatology, quantified through the HAM-D17 scale, with the rising number of chronic conditions. However, the magnitude of this statistically significant result is numerically small, suggesting that the impact of comorbidities on the total depression score is modest in this study group.

The role of calcium in the pathophysiology of affective disorders is multidimensional based on its implications in the functioning of the HPA axis, upregulation of TRP hydroxylase in the serotonin biosynthetic pathway, modulation of dopaminergic homeostasis, and the brain reward circuit [63,64]. Although its absorption and homeostasis are dependent on the action of calcitriol on intestinal and renal receptors [64,65], the mechanism through which calcium intake directly affects the onset of depression remains controversial [64]. While some research, such as the study conducted by Shen et al., indicated a negative relationship between calcium intake and the risk of depression [63], a meta-analysis fails to confirm the presence of a significant association between calcium dietary intake and the incidence of affective disorders [66]. Spearman correlation coefficient revealed an inverse association between total serum calcium concentration and the severity of depressive symptomatology, yet without reaching statistical significance (*p* > 0.05). Regarding the results from ordered probit regression, a significant and negative correlation with the severity of depression, quantified through the BDI-II scale, was demonstrated. The absence of a significant finding for the HAM-D17 scale suggests that this relationship may be specific to the subjective dimension of depressive symptomatology, warranting caution in generalizing this interpretation.

The present study, which primarily aimed to evaluate the relationship between serum vitamin D concentration and the severity of depressive symptomatology, has several strengths that enhance its validity and relevance. First, given the findings obtained through multivariable linear regression analysis, which confirmed the significantly negative association between vitamin D status and the severity of the depressive episode, it was possible to apply the ordered probit regression, enabling the estimation of the probability of membership in the clinical depression severity categories based on vitamin D status and the confounding factors. The complementary use of multivariable linear regression and ordered probit regression constitutes an additional methodological strength, as the convergence of results across both analytical approaches—one treating depression severity as a continuous outcome and the other as an ordinal categorical variable—provides internal validation of the identified association. Second, the study included patients diagnosed with MDD following a clinical assessment conducted by the attending consultant psychiatrist in accordance with the International Classification of Diseases, Tenth Revision (ICD-10) criteria, an aspect that ensured the accuracy of the nosological classification of participants. Third, to quantify the severity of depressive symptomatology, two complementary psychometric scales were simultaneously applied—the BDI-II as a self-report scale [67] and the HAM-D17 as a clinician-administered rating scale [68]—both widely used and validated in the scientific literature. This dual subjective and objective approach minimized the risk of individual reporting errors and consolidated the validity of the observed associations between vitamin D status and depression severity. Fourth, the monocentric study character guaranteed consistent assessment conditions—the same environment and the same methodology uniformly applied to all participants—thus reducing inter-institutional variability. Fifth, vitamin D status was quantified by measuring the serum 25(OH)D concentration, an established biomarker that reflects the combined vitamin D intake from all sources: diet, supplements, and cutaneous synthesis stimulated by UVB radiation [69]. Sixth, the analysis included adjustments for a considerable number of confounding factors encompassing medical history, sociodemographic characteristics, lifestyle, and environmental factors, thereby increasing the robustness of the obtained estimates. Finally, to the best of our knowledge, this is the first study that evaluates the relationship between serum vitamin D concentration and depressive symptomatology in a Romanian population, thereby contributing to filling a gap in the literature regarding this association in the national clinical and demographic contexts.

However, the study has a series of limitations that should be considered when interpreting the results. First, the cross-sectional design does not permit causal inference between vitamin D status and the severity of depressive symptomatology. The reverse causality cannot be excluded, whereby depression leads to reduced sun exposure and poor dietary intake, causing vitamin D deficiency [39]. Observational studies, nonetheless, represent a valuable source of scientific evidence, contributing to the overall evaluation of available data [8]. Second, although the analysis included adjustments for a considerable number of confounding factors, the presence of residual confounding cannot be excluded. It is possible that certain unmeasured covariates, such as physical activity, serum parathyroid hormone (PTH) concentration, smoking, alcohol use, the use of sunscreen products, vitamin D supplementation, or others, may have influenced the results obtained had they been quantified and incorporated into the regression models. Third, the relatively limited sample size represents an important limitation with multiple implications for statistical analysis. Sex could not be included as a covariate in multivariable linear regression and ordered probit regression due to the small number of male participants. Furthermore, the ordered probit regression for the BDI-II scale included a very limited number of subjects in the mild depression category (N = 6), which may affect the stability and robustness of the obtained estimates for this category. These aspects, combined with the monocentric character of the study, may limit the generalizability of the results, both to the general population of patients with MDD in Romania and to other populations. Nevertheless, the post hoc power analysis revealed a very high statistical power for both fully adjusted depression models (99.57% for BDI-II and 98.03% for HAM-D17), indicating that, despite these limitations, the models were robust and the reported associations reliable. Finally, an additional limitation concerns the evaluation of dietary intake of vitamin D, which was estimated through a semi-quantitative frequency questionnaire based on the consumption of vitamin D-containing foods, according to NIH-ODS [47]. Although the calculation methodology was systematic and standardized, the instrument used was not validated on the Romanian population and was based on self-reported dietary intake, potentially introducing estimation errors related to patients’ memory variability and seasonal fluctuations in dietary habits. Similarly, sun exposure was quantified based on self-reported daily sun exposure time, without accounting for factors that influence cutaneous synthesis, such as the sun protection factor (SPF) used, exposed body surface, cutaneous pigmentation, or the angle of incidence of UV radiation relative to season and geographical latitude. This approach may underestimate or overestimate the real contribution of cutaneous synthesis on serum 25(OH)D status.

It should be noted that methodological heterogeneity across studies in the field—including differences in depression assessment tools, 25(OH)D measurement methods, and study design—contributes to the variability of findings reported in the literature. Despite these limitations, the disability associated with MDD represents a significant burden for both public health and modern economies, warranting the continued investigation of therapeutic solutions. Given that currently available therapeutic options remain suboptimal in terms of clinical efficacy, and considering that, to the best of our knowledge, data from Central and Eastern Europe, and particularly from Romania, remain scarce. Epidemiological evidence such as that provided by the present study continues to be examined in order to identify potentially actionable therapeutic targets, which may substantially contribute to optimaizing strategies for the prevention and treatment of MDD.

## 3. Materials and Methods

### 3.1. Study Design

This is a cross-sectional study that primarily aimed to evaluate the relationship between vitamin D status and the severity of depressive symptomatology. The study was conducted between March 2024 and November 2025 at the County Clinical Emergency Hospital of Bihor, Oradea, Romania, and included patients diagnosed with MDD. To quantify the severity of depressive symptomatology, the Beck Depression Inventory-II (BDI-II) and the Hamilton Depression Rating Scale 17 (HAM-D17) were administered. To determine vitamin D status, blood samples were collected for the measurement of the 25(OH)D metabolite. Additionally, information on other relevant covariates with a potential influence on the aforementioned parameters was collected.

The study was reviewed and approved by the Ethics Committee of the Faculty of Medicine and Pharmacy, University of Oradea (Research Ethics Committee Decision No. CEFMF/4, 31 January 2023). Furthermore, the study also received approval from the Ethics Committee of the Bihor County Emergency Clinical Hospital (No. 38797, 18 November 2022) and the endorsement of the Ethical Council of the Bihor County Emergency Clinical Hospital (No. 37935, 11 November 2022). All investigations were conducted in accordance with the Declaration of Helsinki.

### 3.2. Setting and Participants

The study included a total of 90 patients, aged between 34 and 77 years, recruited between March 2024 and November 2025, and diagnosed with MDD by the attending consultant psychiatrist in accordance with ICD-10 criteria.

Inpatients admitted for either continuous or day hospitalization to the Department of Psychiatry at the County Clinical Emergency Hospital of Bihor, aged over 18 years and diagnosed with MDD by the attending physician, were considered eligible. Pregnant and breastfeeding women, individuals with harmful use of alcohol or other substances, and patients with significant cognitive impairment that could interfere with the accurate completion of psychometric assessments were excluded from the study. The sample size was determined by the total number of eligible patients who consented to participate during the data collection period and were enrolled consecutively as they presented to the clinic. A post hoc power analysis was conducted using G*Power (version 3.1.9.7) to evaluate the statistical power achieved by the fully adjusted linear regression models.

In a short interview, the enrollees were informed about the conduct of the study and its objectives, and those who were interested and met the inclusion and exclusion criteria signed an informed consent form. The necessary information and blood samples were collected immediately after consent was obtained, as no follow-up was required due to the cross-sectional study design. All study participants were using antidepressant medication at the time of study enrollment. All study participants were receiving standard antidepressant treatment at the time of enrollment, most commonly selective reuptake inhibitors (SSRIs) or serotonin–norepinephrine reuptake inhibitors (SNRIs). In patients with more severe clinical presentations, concomitant treatment with mood stabilizers, antipsychotics, or benzodiazepines was administered according to standard clinical protocols. Both first-episode and recurrent MDD cases were included in the sample.

### 3.3. Assessments and Measurements

#### 3.3.1. Standardized Interview

Information regarding medical history, sociodemographic characteristics, lifestyle, and environmental factors relevant to the proposed objectives was collected following a standardized interview. In order to avoid the risk of bias, the assessment was conducted under conditions of confidentiality using standardized procedures; prior to its initiation, clear explanations were provided. The included covariates were selected from the scientific literature based on their potential association with vitamin D levels, depressive symptomatology, and their putative role as confounding factors in the relationship between these parameters. Additionally, the covariates were measured using the same instruments and procedures across all subjects, regardless of vitamin D classification group or symptom severity, ensuring the comparability of assessment methods. Missing data were verified, and participants with incomplete data values were excluded to avoid the introduction of further bias.

From the perspective of medical history, information about the number of chronic diseases (0, 1, ≥2), based on patients’ self-reported diagnoses received from their attending physician, and supplemented by the identification of medications brought to the interview, was obtained. Chronic conditions included diabetes mellitus, cardiovascular diseases, dyslipidemia, endocrine disorders, hepatic disorders, neurological disorders, degenerative osteoarticular conditions, chronic respiratory diseases, osteoporosis, and gastrointestinal conditions.

The following sociodemographic characteristics were age (years), sex (F, M), and place of residence (urban, rural). In terms of educational attainment, the subjects were classified into one of two categories: low (no formal education, primary school, or lower secondary school ≤ 8 grades) or medium-to-high (vocational school, high school, or higher education: bachelor’s, master’s, or doctoral degree).

Relevant covariates from the lifestyle and environmental factor categories are sun exposure and the estimated average daily dietary intake of vitamin D. Sun exposure was estimated based on participants’ responses regarding the average daily time spent outdoors. They were classified into one of three categories: <15 min/day, 15–30 min/day, or >30 min/day. The estimated average daily dietary intake of vitamin D (IU/day) was assessed using a semi-quantitative frequency questionnaire evaluating the consumption of vitamin D-containing foods, according to NIH-ODS [47]. Consumption frequencies (never, rare, 1–2 portions/week, 3–5 portions/week, 1 portion/day, 2–3 portions/day, and ≥4 portions/day) were converted into a numerical score (0, 0.5, 1.5, 4, 7, 17.5, and 28). For each food item, the following were calculated: IU of vitamin D per week based on frequency (portions/week) and vitamin D content (IU/portion). Through the summation of IU of vitamin D/week for each food item, the total weekly vitamin D intake was obtained for each study participant. By dividing the total weekly vitamin D intake by 7, the estimated average daily dietary intake of vitamin D (IU/day) was determined per participant.

As a control variable, the analysis accounted for the season of blood withdrawal to reduce the influence of seasonal variation on serum 25(OH)D concentrations. Thus, the season of biological sample collection was dichotomized into a period of high levels of vitamin D (summer time), between March and August, and a period of low levels of vitamin D (winter time), between September and February [56]. 

#### 3.3.2. Anthropometric Measures

Body mass index (BMI) (kg/m^2^) was calculated as the ratio of body weight in kilograms to the square of height in meters, using standardized procedures [70]. Based on BMI values, the subjects were classified into one of six categories: <18.5 (underweight), 18.5–24.99 (healthy weight), 25.00–29.99 (overweight), 30.00–34.99 (class 1 obesity), 35.00–39.99 (class 2 obesity), and ≥40.00 (class 3 obesity) [71].

#### 3.3.3. Psychological Assessment

Data on the severity of the depressive episode were obtained following the administration of two validated scales by the clinical psychologist: the BDI-II and the HAM-D17.

BDI-II is a questionnaire consisting of 21 self-report items that allow for the evaluation of the presence and severity of depressive symptomatology [67]. The validated Romanian version of the questionnaire was used in the study. In terms of standardization, scores ranging from 0 to 13 indicate minimal depression, 14 to 19 indicate mild depression, 20 to 28 indicate moderate depression, and 29 to 63 indicate severe depression [72].

The HAM-D17 scale was developed as an instrument to evaluate the degree of depressive severity based on 17 items that measure the intensity of symptomatology [68]. In contrast to the BDI-II scale, the HAM-D17 is administered by a clinical psychologist during a semi-structured interview. The validated Romanian version of the scale was used in the study. Scores below 7 indicate the absence of depression, scores ranging from 7 to 17 indicate mild depression, scores from 18 to 24 indicate moderate depression, and scores above 25 indicate severe depression [73].

#### 3.3.4. Blood Sample Collection and Laboratory Measurements

Serum vitamin D concentration was determined by measuring the 25(OH)D metabolite from a venous blood sample collected in the morning under overnight fasting conditions. Blood analysis was performed by an accredited laboratory (Bioclinica SRL, Oradea, Romania), using the ARCHITECT 25 OH vitamin D (Abbott Laboratories, Chicago, IL, USA) assay. The chemiluminescence microparticle immunoassay (CMIA) method was used for the quantitative determination of 25(OH)D. This method involves the release of vitamin D from its complex with vitamin D binding protein, followed by its competitive binding to the anti-vitamin D-coated paramagnetic microparticles. A conjugate containing acridinium-labeled vitamin D competes with the analyte from the sample for the antibody binding sites. The generated chemiluminescence signal, automatically quantified as relative light units (RLU) based on a calibration curve, reflects the concentration of 25(OH)D in the sample. In terms of interpretation, a metabolite value of <20 ng/mL is considered deficiency, values between 20 and <30 ng/mL indicate insufficiency, and values between 30 and 100 ng/mL are classified as optimal [74].

Total serum calcium was quantified from a venous blood sample collected in the morning under overnight fasting conditions. After collection, total serum calcium was measured from the blood serum by spectrophotometry using the automated system Alinity c (Abbott Ireland, Dublin, Ireland). This method relies on the reaction between calcium and Arsenazo III in an acidic solution, resulting in the formation of a blue–purple complex. The analysis was performed at the County Clinical Emergency Hospital of Bihor laboratory (Oradea, Romania). The results were expressed in mg/dL, and the optimal values range between 8.4 and 10.2 mg/dL [75].

### 3.4. Statistical Analysis

STROBE criteria were strictly adhered to throughout the conduct of the study [76]. For sample characterization, continuous variables with a normal distribution were presented as mean ± standard deviation (SD), while continuous variables with a non-normal distribution were presented as median, interquartile range (IQR), and range (min–max). Categorical variables were described as number (N) and percentage (%). The normality of the distribution was assessed using the Shapiro–Wilk test (*p* > 0.05 indicating a normal distribution) (Table 1).

In the preliminary stage of the multivariable analysis, a univariable analysis was performed to explore the bivariate correlations between each of the dependent variables (BDI-II score, HAM-D17 score, and serum vitamin D concentration) and independent variables. For continuous variables with a normal distribution, between-group comparisons were performed using the independent samples *t*-test (for dichotomous variables) and one-way ANOVA (for variables with more than two categories), with post hoc comparisons using the Dunn–Sidak correction for multiple comparisons. For continuous variables with a non-normal distribution, the corresponding non-parametric alternatives were used: the Mann–Whitney U test and the Kruskal–Wallis test, with post hoc comparisons using the Dunn test with Bonferroni correction. To evaluate the association between continuous variables, the Spearman correlation coefficients were used. The convergent validity of the two instruments used to assess depression was verified by analyzing the association between the total scores obtained on each scale using the Spearman correlation coefficient (Supplementary Tables S1–S4).

The link between serum 25(OH)D concentration and the severity of depressive symptoms was individually examined for each rating scale using multivariable linear regression analysis. Progressive adjustment models were constructed to examine the successive impact of potential confounding factors. The unadjusted model (Model 0) included only serum vitamin D concentration. Model 1 was additionally adjusted for age and the season of blood withdrawal. Model 2 further incorporated lifestyle-related factors (place of residence, education, BMI, the estimated average daily dietary intake of vitamin D, and sun exposure), and Model 3 additionally adjusted for clinical variables (total serum calcium and the number of chronic diseases). The covariates were selected from the scientific literature. For each model, the standardized coefficient (β), unstandardized coefficient (B), and corresponding *p*-values were reported. The stages of multivariable analysis were consistently and independently applied for both scales (the BDI-II and the HAM-D17), thus ensuring a robust and comparable evaluation of the impact of 25(OH)D concentrations on all dimensions of the symptomatology studied (Table 2).

Given that the multivariable linear regression analysis confirmed the existence of a significant association between vitamin D status and the severity of the depressive episode, ordered probit regression (oprobit Stata command) was additionally applied separately for each assessment scale—the BDI-II and the HAM-D17. The objective of this analysis was to estimate the probability of membership in the clinical depression severity categories based on vitamin D status and the covariates included in the model. The dependent variable was the depression severity category defined by the validated cut-off values of each scale, and the primary independent variable was serum 25(OH)D concentration. The models were adjusted for the same covariates included in the multivariable linear regression analysis. The coefficients and the cut-points were derived using the maximum likelihood estimation method (the model parametrization does not include a constant, as its effect is absorbed into the cut-points). Standard errors were estimated using the robust method (robust standard errors) to account for potential heteroscedasticity. For categorical variables, a reference category was omitted from the model, against which the coefficients for the remaining categories were interpreted (Table 3 and Table 4). Since the raw predicted values generated by the model failed to correspond to the range of values of the original dependent variables (as is expected when applying ordered probit regression), and given that negative index values lack direct clinical interpretability, these were subjected to a normalization procedure using the min–max normalization method, resulting in values within the range of [0–1]. Both the predicted linear index and the model-derived cut-points were normalized using the same min–max transformation and identical reference minimum and maximum values, thereby preserving the relative ordering of the original cut-points. To align the rescaled index with the number of severity categories defined for each scale, the normalized values were subsequently multiplied by the number of categories—4 for BDI-II and 3 for HAM-D17—yielding a rescaled index ranging from 0 to 4 for BDI-II and from 0 to 3 for HAM-D17. The rescaled values were subsequently recoded into an ordinal categorical variable with four levels for the BDI-II (value 1 for the minimal category, value 2 for the mild category, value 3 for the moderate category, value 4 for the severe category), and three levels for the HAM-D17 (value 1 for the mild category, value 2 for the moderate category, value 3 for the severe category). The following cut-points were applied: for the BDI-II—the minimal category for values ≤ 1.80, the mild category for values within the range (1.80–2.50], the moderate category for values within the range (2.50–3.10], and the severe category for values > 3.10; for the HAM-D17—the mild category for values ≤ 1.77 (7), the moderate category for values within the range (1.77 (7)–2.470], and the severe category for values > 2.470. Each step of this process—raw values, normalized values, and final clinical categories—was examined through graphical representation using histograms (Figure 1, Figure 2, Figure 3, Figure 4, Figure 5 and Figure 6). The categories thus obtained were compared with the observed severity categories by constructing a classification table, and the model global accuracy was calculated as the proportion of correctly classified cases out of the total number of participants (Table 5 and Table 6).

The individuals with incomplete data were excluded from the study. A *p*-value < 0.05 was considered statistically significant. The analysis was performed using the STATA software, StataCorp 19.5 SE (4905 Lakeway Drive, College Station, TX, USA).

## 4. Conclusions

In conclusion, the study provides relevant information regarding the relationship between serum vitamin D concentration and the severity of depressive symptomatology in a sample of adult patients from Romania diagnosed with MDD. The statistical analyses conducted independently for the two depression rating scales (the BDI-II scale and the HAM-D17 scale) highlighted a negative correlation between vitamin D status and depressive symptomatology, a relationship that remained robust even after adjusting the regression models for diverse covariates. Furthermore, the findings of ordered probit regression analysis suggested that achieving sufficient vitamin D levels is associated with a significantly lower probability of belonging to a higher depression severity category compared with deficiency, indicating that the clinically relevant threshold lies at the transition between deficiency and sufficiency.

Given that cross-sectional studies, by the nature of their design, do not allow for the establishment of a causal relationship, elucidating the direction of this association requires the conducting of longitudinal studies and randomized clinical trials, characterized by more homogeneous methodologies and designed to integrate the clinical and biological heterogeneity of depressive disorder. Through such research, it may be determined if vitamin D deficiency constitutes a consequence or a causal factor in depression, and if its supplementation may prove beneficial in alleviating depressive symptomatology.

## Figures and Tables

**Table 1:** Descriptive analysis of sociodemographic, clinical, and biological characteristics (N = 90).

Variable	Value
Age (years), median (IQR), min–max	56 (8), 34–77
30–49, N (%)	16 (17.78)
50–59, N (%)	45 (50.00)
60+, N (%)	29 (32.22)
Sex, N (%)	
F	85 (94.44)
M	5 (5.56)
BMI (kg/m^2^), mean ± SD	30.04 ± 6.172
<18.5, N (%)	2 (2.22)
18.5–24.99, N (%)	14 (15.56)
25.00–29.99, N (%)	33 (36.67)
30.00–34.99, N (%)	25 (27.78)
35.00–39.99, N (%)	11 (12.22)
≥40.00, N (%)	5 (5.56)
Place of residence, N (%)	
Rural	52 (57.78)
Urban	38 (42.22)
Education, N (%)	
Low	13 (14.44)
Medium–high	77 (85.56)
Season of blood withdrawal, N (%)	
Summer time	64 (71.11)
Winter time	26 (28.89)
Sun exposure (min/day), N (%)	
<15	22 (24.44)
15–30	17 (18.89)
>30	51 (56.67)
Estimated average daily dietary intake of vitamin D (IU/day), median (IQR), min–max	140.28 (154), 6.14–1610.29
Number of chronic diseases, N (%)	
0	23 (25.56)
1	18 (20.00)
≥2	49 (54.44)
Total serum calcium (mg/dL), mean ± SD	9.04 ± 0.526
BDI-II, median (IQR), min–max	25.00 (13.75), 5–63
0–13, N (%)	11 (12.22)
14–19, N (%)	6 (6.67)
20–28, N (%)	36 (40.00)
29–63, N (%)	37 (41.11)
HAM-D17, mean ± SD	20.50 ± 4.786
7–17, N (%)	16 (17.78)
18–24, N (%)	55 (61.11)
≥25, N (%)	19 (21.11)
Serum 25(OH)D (ng/mL), mean ± SD	22.77 ± 9.058
<20, N (%)	35 (38.89)
20–<30, N (%)	38 (42.22)
30–100, N (%)	17 (18.89)

Abbreviations: 25(OH)D = 25-hydroxyvitamin D; BDI-II = Beck Depression Inventory-II; BMI = body mass index; F = female; HAM-D17 = Hamilton Depression Rating Scale 17; IQR = interquartile range; IU = international units; M = male; N = number; SD = standard deviation.

**Table 2:** Multivariable linear regression analysis: association between serum 25(OH)D concentrations and depression severity (Models 0–3).

Serum 25(OH)D (ng/mL)	BDI-II											
	Model 0			Model 1			Model 2			Model 3		
	*B*	*β*	*p*-Value *	*B*	*β*	*p*-Value *	*B*	*β*	*p*-Value *	*B*	*β*	*p*-Value *
**Continuous level**	−0.3170444	−0.2621328	0.007	−0.3176379	−0.2626235	0.006	−0.2387353	−0.1973867	0.025	−0.2110473	−0.1744942	0.050
	**HAM-D17**											
	**Model 0**			**Model 1**			**Model 2**			**Model 3**		
	***B***	***β***	***p*** -Value *	***B***	***β***	***p*** -Value *	***B***	***β***	***p*** -Value *	***B***	***β***	***p*** -Value *
**Continuous level**	−0.1495026	−0.2829461	0.002	−0.146535	−0.2773298	0.003	−0.1222592	−0.2313858	0.010	−0.1153659	−0.2183397	0.018

Abbreviations: 25(OH)D = 25-hydroxyvitamin D; BDI-II = Beck Depression Inventory-II; HAM-D17 = Hamilton Depression Rating Scale 17. B = unstandardized beta (β). Model 0: unadjusted. Model 1: adjusted for age (continuous) and season of blood withdrawal (summer time, winter time). Model 2: additionally adjusted for education (low, medium-to-high), place of residence (rural, urban), the estimated average daily dietary intake of vitamin D (IU/day, continuous), BMI (kg/m^2^, continuous) and sun exposure (min/day, <15, 15–30, >30). Model 3: additionally adjusted for number of chronic diseases (0, 1, ≥2) and total serum calcium (mg/dL, continuous). Note: post hoc power analysis for the fully adjusted model (Model 3) indicated an achieved statistical power of 99.57% for the BDI-II model and 98.03% for the HAM-D17 model (α = 0.05), based on the observed effect sizes and final sample size (N = 90). * Statistically significant < 0.05.

**Table 3:** Ordered probit regression analysis of depression severity assessed by BDI-II.

numBDI-II	Coefficient	Robust Std. Err.	*p*-Value
numAGE			
50–59	0.7329627	0.2940305	0.013
60+	−0.0009885	0.2814682	0.997
num25OHD			
insuf	−0.2868912	0.1939367	0.139
suf	−0.6855429	0.2349612	0.004
numISE			
15–30	−0.1272801	0.2644052	0.630
>30	0.0116318	0.235147	0.961
numSOBW			
Winter.Time	0.0531621	0.2097905	0.800
numRSD			
U	−0.2542674	0.2061857	0.218
numOCD			
1	0.4196731	0.2636917	0.111
≥2	0.2663627	0.2424683	0.272
numED			
MED-HIGH	−0.6392058	0.3109966	0.040
tsca	−0.3741122	0.1786114	0.036
bmi	−0.0127006	0.0153929	0.409
dvdi	0.0003804	0.0002965	0.199
numBDI-II	1	(offset)	
/cut1	−3.084909	1.68131	
/cut2	−2.360221	1.712636	
/cut3	−0.3766894	1.674623	

Reference categories: numAGE—30–49; num25(OH)D—deficiency; numISE—<15; numSOBW—Summer.Time; RSD—rural; numOCD—0; numED—low. Abbreviations: 25(OH)D = 25-hydroxyvitamin D; BDI-II = Beck Depression Inventory-II; BMI = body mass index; cut1, cut2, cut3 = cut-points estimated by maximum likelihood; dvdi = the estimated average daily dietary intake of vitamin D; ED = education; insuf = insufficiency; ISE = sun exposure; MED-HIGH = medium-to-high; OCD = number of chronic diseases; RSD = place of residence; SOBW = season of blood withdrawal; suf = sufficiency; std.err. = standard error; tsca = total serum calcium; U = urban.

## Data Availability

The data presented in this study are not publicly available due to privacy and ethical restrictions related to patient confidentiality.

## Supplementary Materials

The following supporting information can be downloaded at: https://www.mdpi.com/article/10.3390/ijms27188192/s1.
