# Supplementary material for: The Association Between Vitamin D Status and Depression Severity in Adults with Major Depressive Disorder: A Cross-Sectional Study
Source: Int J Mol Sci. 2026 Sep 15;27(18):8192. doi: 10.3390/ijms27188192 (PMC13607366; doi:10.3390/ijms27188192)
Supplement: Supplementary file 1 [file ijms-27-08192-s001.zip › ijms-4496516-supplementary.pdf]

Supplementary Table S1: Bivariate analysis of associations between serum 25(OH)D concentrations and study covariates

**Serum 25(OH)D (ng/mL)**								
**Variable**	**Pairwise test *p*-values**						**Mean ± SD**	**N (%)**
**Age (years)***	**30-49**		**50-59**		**60+**			
**30-49**	1.000		**< 0.0001**		0.408		20.54 ± 8.458	16 (17.78)
**50-59**	**< 0.0001**		1.000		**< 0.0001**		25.58 ± 9.155	45 (50.00)
**60+**	0.408		**< 0.0001**		1.000		19.64 ± 8.066	29 (32.22)
**Sex****	**F**			**M**				
**F**	1.000			0.887			22.78 ± 9.254	85 (94.44)
**M**	0.887			1.000			22.56 ± 5.209	5 (5.56)
**BMI (kg/m^2)***	**< 18.5**	**18.5–24.99**	**25.00–29.99**	**30.00–34.99**	**35.00–39.99**	**≥ 40.00**		
**< 18.5**	1.000	**< 0.0001**	**< 0.0001**	**< 0.0001**	0.000	0.000	9.85 ± 3.748	2 (2.22)
**18.5–24.99**	**< 0.0001**	1.000	0.067	0.084	0.291	0.700	21.95 ± 7.456	14 (15.56)
**25.00–29.99**	**< 0.0001**	0.067	1.000	0.969	0.005	0.102	24.00 ± 9.455	33 (36.67)
**30.00–34.99**	**< 0.0001**	0.084	0.969	1.000	0.007	0.113	23.96 ± 9.330	25 (27.78)
**35.00–39.99**	0.000	0.291	0.005	0.007	1.000	0.675	20.47 ± 8.480	11 (12.22)
**≥ 40.00**	0.000	0.700	0.102	0.113	0.675	1.000	21.25 ± 9.964	5 (5.56)
**Residence****	**Rural**			**Urban**				
**Rural**	1.000			0.170			23.20 ± 8.557	52 (57.78)
**Urban**	0.170			1.000			22.18 ± 9.787	38 (42.22)
**Education****	**Low**			**Medium-high**				
**Low**	1.000			**< 0.0001**			16.74 ± 7.943	13 (14.44)
**Medium-high**	**< 0.0001**			1.000			23.79 ± 8.880	77 (85.56)
**Season of blood withdrawal****	**Summer time**			**Winter time**				
**Summer time**	1.000			0.668			22.87 ± 9.276	64 (71.11)
**Winter time**	0.668			1.000			22.53 ± 8.669	26 (28.89)
**Sun exposure (min/day)***	**< 15**		**15-30**		**> 30**			
**< 15**	1.000		0.566		0.002		21.03 ± 8.362	22 (24.44)
**15-30**	0.566		1.000		0.026		21.68 ± 10.961	17 (18.89)
**> 30**	0.002		0.026		1.000		23.88 ± 8.673	51 (56.67)

Serum 25(OH)D (ng/mL)						
**Variable**	**Pairwise test *p*-values**				**Mean ± SD**	**N (%)**
**Number of chronic diseases***	**0**	**1**	**≥ 2**			
**0**	1.000	0.642	0.004		24.31 ± 8.506	23 (25.56)
**1**	0.642	1.000	0.028		23.80 ± 6.933	18 (20.00)
**≥ 2**	0.004	0.028	1.000		21.67 ± 9.958	49 (54.44)
**25(OH)D***	**< 20**	**20 - < 30**	**30-100**			
**< 20**	1.000	**< 0.0001**	**< 0.0001**		13.81 ± 4.249	35 (38.89)
**20 - < 30**	**< 0.0001**	1.000	**< 0.0001**		25.24 ± 2.629	38 (42.22)
**30-100**	**< 0.0001**	**< 0.0001**	1.000		35.72 ± 5.810	17 (18.89)
**BDI-II***	**0-13**	**14-19**	**20-28**	**29-63**		
**0-13**	1.000	**< 0.0001**	0.901	0.020	23.38 ± 9.061	11 (12.22)
**14-19**	**< 0.0001**	1.000	**< 0.0001**	**< 0.0001**	30.86 ± 5.759	6 (6.67)
**20-28**	0.901	**< 0.0001**	1.000	0.000	23.53 ± 10.272	36 (40.00)
**29-63**	0.020	**< 0.0001**	0.000	1.000	20.54 ± 7.493	37 (41.11)
**HAM-D17***	**7-17**	**18-24**	**≥ 25**			
**7-17**	1.000	**< 0.0001**	**< 0.0001**		26.64 ± 10.880	16 (17.78)
**18-24**	**< 0.0001**	1.000	0.019		22.50 ± 9.173	55 (61.11)
**≥ 25**	**< 0.0001**	0.019	1.000		20.30 ± 5.881	19 (21.11)

Abbreviations: 25(OH)D = 25-hydroxyvitamin D; BDI-II = Beck Depression Inventory-II; BMI = body mass index; F = female; HAM-D17 = Hamilton Depression Rating Scale 17; M = male; N = number; SD = standard deviation.

For normally distributed continuous data, *p*-values were determined using parametric tests: the independent t-test** for dichotomous variables and one-way ANOVA* for polytomous variables.

Supplementary Table S2: Bivariate analysis of associations between HAM-D17 scores and study covariates

HAM-D17					
**Variable**	**Pairwise test *p*-values**			**Mean ± SD**	**N (%)**
**Age (years)***	**30-49**	**50-59**	**60+**		
**30-49**	1.000	0.003	0.082	19.19 ± 5.683	16 (17.78)
**50-59**	0.003	1.000	0.154	21.07 ± 4.550	45 (50.00)
**60+**	0.082	0.154	1.000	20.34 ± 4.631	29 (32.22)
**Sex****	**F**	**M**			

HAM-D17								
**Variable**	**Pairwise test *p*-values**						**Mean ± SD**	**N (%)**
**F**	1.000			0.233			20.44 ± 4.745	85 (94.44)
**M**	0.233			1.000			21.60 ± 5.941	5 (5.56)
**BMI (kg/m^2)***	**< 18.5**	**18.5–24.99**	**25.00–29.99**	**30.00–34.99**	**35.00-39.99**	**≥ 40.00**		
**< 18.5**	1.000	0.101	0.397	0.410	0.538	0.177	22.00 ± 5.657	2 (2.22)
**18.5–24.99**	0.101	1.000	0.050	0.056	0.057	0.825	19.36 ± 3.272	14 (15.56)
**25.00–29.99**	0.397	0.050	1.000	0.967	0.680	0.280	20.70 ± 4.720	33 (36.67)
**30.00–34.99**	0.410	0.056	0.967	1.000	0.714	0.280	20.72 ± 4.979	25 (27.78)
**35.00–39.99**	0.538	0.057	0.680	0.714	1.000	0.221	21.00 ± 4.539	11 (12.22)
**≥ 40.00**	0.177	0.825	0.280	0.280	0.221	1.000	19.60 ± 8.961	5 (5.56)
**Residence****	**Rural**			**Urban**				
**Rural**	1.000			0.364			20.67 ± 5.075	52 (57.78)
**Urban**	0.364			1.000			20.26 ± 4.415	38 (42.22)
**Education****	**Low**			**Medium-high**				
**Low**	1.000			0.000			22.62 ± 4.154	13 (14.44)
**Medium-high**	0.000			1.000			20.14 ± 4.817	77 (85.56)
**Season of blood withdrawal****	**Summer time**			**Winter time**				
**Summer time**	1.000			0.007			20.11 ± 5.074	64 (71.11)
**Winter time**	0.007			1.000			21.46 ± 3.911	26 (28.89)
**Sun exposure (min/day)***	**< 15**		**15-30**		**> 30**			
**< 15**	1.000		0.068		**< 0.0001**		22.32 ± 4.347	22 (24.44)
**15-30**	0.068		1.000		0.012		21.06 ± 5.093	17 (18.89)
**> 30**	**< 0.0001**		0.012		1.000		19.53 ± 4.692	51 (56.67)
**Number of chronic diseases***	**0**		**1**		**≥ 2**			
**0**	1.000		0.813		0.003		19.57 ± 4.785	23 (25.56)
**1**	0.813		1.000		0.012		19.72 ± 3.816	18 (20.00)
**≥ 2**	0.003		0.012		1.000		21.22 ± 5.063	49 (54.44)
**25(OH)D***	**< 20**		**20 - < 30**		**30-100**			
**< 20**	1.000		0.261		**< 0.0001**		21.40 ± 5.013	35 (38.89)
**20 - < 30**	0.261		1.000		**< 0.0001**		20.84 ± 4.284	38 (42.22)

HAM-D17						
**Variable**	**Pairwise test *p*-values**				**Mean ± SD**	**N (%)**
**30-100**	**< 0.0001**	**< 0.0001**	1.000		17.88 ± 4.715	17 (18.89)
**BDI-II***	**0-13**	**14-19**	**20-28**	**29-63**		
**0-13**	1.000	0.039	**< 0.0001**	**< 0.0001**	15.09 ± 3.807	11 (12.22)
**14-19**	0.039	1.000	0.015	**< 0.0001**	17.33 ± 4.633	6 (6.67)
**20-28**	**< 0.0001**	0.015	1.000	**< 0.0001**	19.64 ± 3.373	36 (40.00)
**29-63**	**< 0.0001**	**< 0.0001**	**< 0.0001**	1.000	23.46 ± 4.266	37 (41.11)
**HAM-D17***	**7-17**	**18-24**	**≥ 25**			
**7-17**	1.000	**< 0.0001**	**< 0.0001**		13.38 ± 2.391	16 (17.78)
**18-24**	**< 0.0001**	1.000	**< 0.0001**		20.29 ± 2.105	55 (61.11)
**≥ 25**	**< 0.0001**	**< 0.0001**	1.000		27.11 ± 1.969	19 (21.11)

Abbreviations: 25(OH)D = 25-hydroxyvitamin D; BDI-II = Beck Depression Inventory-II; BMI = body mass index; F = female; HAM-D17 = Hamilton Depression Rating Scale 17; M = male; N = number; SD = standard deviation.

For normally distributed continuous data, *p*-values were determined using parametric tests: the independent t-test** for dichotomous variables and one-way ANOVA* for polytomous variables.

Supplementary Table S3: Bivariate analysis of associations between BDI-II scores and study covariates

BDI-II								
**Variable**	**Pairwise test *p*-values**						**Median (Q1-Q3), min-max**	**N (%)**
**Age (years)***	**30-49**	**50-59**	**60+**					
**30-49**	1.000	0.187	0.842				24.00 (15.25-30.50), 6-63	16 (17.78)
**50-59**	0.187	1.000	0.176				29.00 (21.00-34.00), 8-57	45 (50.00)
**60+**	0.842	0.176	1.000				22.00 (20.00-34.00), 5-49	29 (32.22)
**Sex****	**F**	**M**						
**F**	1.000	0.665					25.00 (20.00-33.00), 5-63	85 (94.44)
**M**	0.665	1.000					32.00 (20.00-35.00), 10-48	5 (5.56)
**BMI (kg/m^2)***	**< 18.5**	**18.5–24.99**	**25.00–29.99**	**30.00-34.99**	**35.00–39.99**	**≥ 40.00**		
**< 18.5**	1.000	0.431	0.409	0.640	0.831	0.273	30.00 (26.00-34.00), 22-38	2 (2.22)
**18.5–24.99**	0.431	1.000	0.987	0.450	0.284	0.538	25.50 (20.50-31.00), 5-35	14 (15.56)
**25.00–29.99**	0.409	0.987	1.000	0.332	0.209	0.510	22.00 (20.00-33.00), 10-63	33 (36.67)
**30.00–34.99**	0.640	0.450	0.332	1.000	0.619	0.242	26.00 (22.00-34.00), 8-57	25 (27.78)

BDI-II								
**Variable**	**Pairwise test *p*-values**						**Median (Q1-Q3), min-max**	**N (%)**
**35.00–39.99**	0.831	0.284	0.209	0.619	1.000	0.163	32.00 (20.00-37.00), 20-39	11 (12.22)
**≥ 40.00**	0.273	0.538	0.510	0.242	0.163	1.000	24.00 (8.00-28.00), 6-34	5 (5.56)
**Residence****	**Rural**	**Urban**						
**Rural**	1.000	0.029					29.00 (20.00-34.25), 6-63	52 (57.78)
**Urban**	0.029	1.000					22.00 (20.00-28.75), 5-57	38 (42.22)
**Education****	**Low**	**Medium-high**						
**Low**	1.000	0.015					34.00 (25.00-41.00), 19-63	13 (14.44)
**Medium-high**	0.015	1.000					23.00 (20.00-32.00), 5-49	77 (85.56)
**Season of blood withdrawal****	**Summer time**	**Winter time**						
**Summer time**	1.000	0.333					25.50 (20.00-35.00), 6-63	64 (71.11)
**Winter time**	0.333	1.000					24.50 (20.00-29.75), 5-36	26 (28.89)
**Sun exposure (min/day)***	**< 15**	**15-30**	**> 30**					
**< 15**	1.000	0.681	0.903				25.00 (20.00-36.00), 5-49	22 (24.44)
**15-30**	0.681	1.000	0.716				22.00 (20.00-29.00), 16-63	17 (18.89)
**> 30**	0.903	0.716	1.000				25.00 (20.00-33.00), 6-57	51 (56.67)
**Number of chronic diseases***	**0**	**1**	**≥ 2**					
**0**	1.000	0.319	0.053				22.00 (18.00-29.00), 8-46	23 (25.56)
**1**	0.319	1.000	0.522				26.50 (20.50-32.75), 5-39	18 (20.00)
**≥ 2**	0.053	0.522	1.000				26.00 (20.00-36.00), 8-63	49 (54.44)
**25(OH)D***	**< 20**	**20 - < 30**	**30-100**					
**< 20**	1.000	0.453	0.017				25.00 (20.00-36.00), 10-63	35 (38.89)
**20 - < 30**	0.453	1.000	0.069				27.00 (20.00-31.75), 6-48	38 (42.22)
**30-100**	0.017	0.069	1.000				20.00 (15.00-26.00), 5-37	17 (18.89)
**BDI-II***	**0-13**	**14-19**	**20-28**	**29-63**				
**0-13**	1.000	0.521	0.001	**< 0.0001**			10.00 (8.00-11.00), 5-13	11 (12.22)
**14-19**	0.521	1.000	0.068	**< 0.0001**			16.00 (15.25-17.50), 15-19	6 (6.67)
**20-28**	0.001	0.068	1.000	**< 0.0001**			22.00 (20.00-24.25), 20-28	36 (40.00)
**29-63**	**< 0.0001**	**< 0.0001**	**< 0.0001**	1.000			35.00 (32.00-38.00), 29-63	37 (41.11)

BDI-II					
**Variable**	**Pairwise test *p*-values**			**Median (Q1-Q3), min-max**	**N (%)**
**HAM-D17***	**7-17**	**18-24**	**≥ 25**		
**7-17**	1.000	0.001	**< 0.0001**	15.50 (10.00-20.50), 6-38	16 (17.78)
**18-24**	0.001	1.000	0.001	25.00 (20.00-32.50), 5-46	55 (61.11)
**≥ 25**	**< 0.0001**	0.001	1.000	34.00 (29.00-38.00), 20-63	19 (21.11)

Abbreviations: 25(OH)D = 25-hydroxyvitamin D; BDI-II = Beck Depression Inventory-II; BMI = body mass index; F = female; HAM-D17 = Hamilton Depression Rating Scale 17; M = male; N = number; SD = standard deviation.

For non-normally distributed continuous data, *p*-values were determined using non-parametric tests: Mann-Whitney** for dichotomous variables and Kruskal-Wallis* for polytomous variables.

Supplementary Table S4: Spearman correlation coefficients between continuous variables

**Variables**	**Age (years) (A)**		**Serum 25(OH)D (ng/mL) (N)**		**BDI-II (A)**		**HAM-D17 (N)**		**Total serum calcium (mg/dL) (N)**		**The estimated average daily dietary intake of vitamin D (IU/day) (A)**		**BMI (kg/m^2) (N)**	
	**r_S**	***p*-value**	**r_S**	***p*-value**	**r_S**	***p*-value**	**r_S**	***p*-value**	**r_S**	***p*-value**	**r_S**	***p*-value**	**r_S**	***p*-value**
**Age (years) (A)**	1	0	-0.069	0.518	0.041	0.702	0.095	0.370	0.127	0.233	-0.015	0.888	0.026	0.805
**Serum 25(OH)D (ng/mL) (N)**	-0.069	0.518	1	0	-0.256	0.015	-0.211	0.046	0.066	0.534	0.082	0.443	-0.018	0.863
**BDI-II (A)**	0.041	0.702	-0.256	0.015	1	0	0.583	< 0.0001	-0.143	0.179	-0.191	0.071	0.128	0.228
**HAM-D17 (N)**	0.095	0.370	-0.211	0.046	0.583	< 0.0001	1	0	-0.170	0.110	-0.242	0.022	0.111	0.297
**Total serum calcium**	0.127	0.233	0.066	0.534	-0.143	0.179	-0.170	0.110	1	0	0.056	0.596	-0.152	0.154

Variables	Age (years) (A)		Serum 25(OH)D (ng/mL) (N)		BDI-II (A)		HAM-D17 (N)		Total serum calcium (mg/dL) (N)		The estimated average daily dietary intake of vitamin D (IU/day) (A)		BMI (kg/m^2) (N)	
	r_S	*p*-value	r_S	*p*-value	r_S	*p*-value	r_S	*p*-value	r_S	*p*-value	r_S	*p*-value	r_S	*p*-value
**(mg/dL) (N)**														
**The estimated average daily dietary intake of vitamin D (IU/day) (A)**	-0.015	0.888	0.082	0.443	-0.191	0.071	-0.242	0.022	0.056	0.596	1	0	0.062	0.559
**BMI (kg/m^2) (N)**	0.026	0.805	-0.018	0.863	0.128	0.228	0.111	0.297	-0.152	0.154	0.062	0.559	1	0

Abbreviations: 25(OH)D = 25-hydroxyvitamin D; A = non-normally distributed; BDI-II = Beck Depression Inventory-II; BMI = body mass index; HAM-D17 = Hamilton Depression Rating Scale 17; IU = international units; N = normally distributed, r_S = Spearman's rank correlation coefficient.
